# Cross-cultural adaptation and initial psychometric evaluation of the fruit and vegetable self-efficacy scales among secondary school adolescents in Selangor, Malaysia

**DOI:** 10.3389/fpubh.2026.1920784

**Published:** 2026-09-29

**Authors:** Zanariah Zaini, Nursyazwani Jabir, Arimi Fitri Mat Ludin, Ruzita Abd Talib, Ismarulyusda Ishak

**Affiliations:** 1 Centre for Toxicology and Health Risk, Faculty of Health Sciences, Universiti Kebangsaan Malaysia, Kuala Lumpur, Malaysia; 2 Institute for Health Behavioural Research, National Institutes of Health, Ministry of Health, Shah Alam, Malaysia; 3 Centre for Healthy Ageing and Wellness, Faculty of Health Sciences, Universiti Kebangsaan Malaysia, Kuala Lumpur, Malaysia; 4 Centre for Community Health Studies, Faculty of Health Sciences, Universiti Kebangsaan Malaysia, Kuala Lumpur, Malaysia

**Keywords:** adolescents, cross-cultural adaptation, fruit and vegetable intake, questionnaire adaptation, self-efficacy

## Abstract

Fruit and vegetable self-efficacy may influence adolescent dietary behavior, yet culturally adapted measures for Malaysian adolescents remain limited. This study aimed to cross-culturally adapt the 17-item Fruit Self-Efficacy (FSE) and Vegetable Self-Efficacy (VSE) scales into Malay and obtain initial evidence on their measurement properties. The study comprised two sequential phases. Phase 1 involved forward and backward translation, cognitive debriefing with five adolescents, linguistic equivalence assessment by six experts, content validity assessment by nine experts, qualitative and quantitative face validity assessment involving 15 unique adolescents, and pretesting with 37 adolescents. Phase 2 comprised an initial psychometric evaluation among 128 adolescents from four classes in one purposively selected public secondary school in Selangor. The adaptation process included age-appropriate refinements to the linguistic and contextual aspects of fruit and vegetable preparation, snacks, social occasions, serving terminology, and meal contexts. In particular, “pieces of fruit” was adapted to “servings of fruit” and supported by a pictorial serving guide, while “hot meal” was contextualized as “main meal.” All source items met the prespecified content-validity criteria. Scale-level content-validity indices were 0.99 for FSE and 0.95 for VSE, while scale-level face-validity indices ranged from 0.95 to 0.97. One FSE item with an initial clarity index below the criterion was revised using concrete preparation examples and reassessed. In the separate psychometric sample, the prespecified one-factor FSE model showed acceptable fit (CFI = 0.974, TLI = 0.970, RMSEA = 0.069, SRMR = 0.061), although pronounced item-level ceiling effects were observed. The prespecified VSE model showed substantial misfit (RMSEA = 0.136, SRMR = 0.099), and its structural validity was not established. A same-sample *post hoc* specification with six correlated residuals remained hypothesis-generating because its RMSEA was 0.094. The multistage process produced linguistically and culturally adapted FSE and VSE scales that met the prespecified content and face-validity criteria among the expert and adolescent samples assessed. However, evidence supporting the one-factor structure was stronger for FSE than VSE. Further cognitive investigation and adequately powered, independent multisite evaluation across diverse ethnic and age groups are required before broader use.

## 1 Introduction

A growing body of evidence indicates that self-efficacy (SE) is a key determinant of fruit and vegetable (FV) consumption among adolescents. Rooted in Social Cognitive Theory (SCT), SE refers to an individual’s belief in their capability to perform specific behaviors under varying conditions and in the presence of situational barriers (1). In dietary behavior research, SE reflects adolescents’ confidence in their ability to select, prepare, and consume fruits and vegetables despite environmental, social, or personal challenges (2–4). Studies consistently demonstrate that adolescents with higher dietary SE are more likely to achieve recommended FV intake levels. Furthermore, SE mediates the relationship between knowledge, environmental exposure, and actual dietary behavior, making it a central mechanism in health behavior change and maintenance (5–7).

Importantly, SE is behavior-specific and context-dependent. Confidence in consuming fruits may differ from confidence in consuming vegetables across varying meal situations, social settings, and environmental constraints (1, 8, 9). Consequently, domain-specific measures such as FVSE provide more accurate predictions of dietary behavior than general SE constructs (10–12). Theoretical extensions, such as the Health Action Process Approach (HAPA), further propose that SE operates across multiple phases of behavior change, including behavioral initiation, maintenance, and recovery from setbacks, each of which requires distinct competencies and confidence levels (13, 14). Together, these theoretical perspectives highlight the importance of developing contextually relevant instruments that capture adolescents’ real-life dietary experiences, sociocultural influences, and perceived barriers to FV consumption.

Despite its theoretical importance, the measurement of FVSE remains inconsistent across studies, with substantial variation in conceptual definitions, item structures, and contextual relevance. Several instruments developed in Western populations demonstrate acceptable psychometric properties, including high internal consistency and construct validity (9, 15). However, many existing measures assess general dietary SE rather than confidence toward specific fruit and vegetable consumption behaviors across realistic adolescent contexts.

International psychometric studies demonstrate that nutrition-related self-efficacy instruments may not retain identical item performance when applied across cultural and demographic contexts. Although the Dutch FSE and VSE instruments showed unidimensional structures and high internal consistency, these findings establish validity only within the population in which they were evaluated (10). Subsequent assessments of related child and adolescent measures have revealed limitations that were not apparent from reliability coefficients alone. Among American children, Watson et al. (16) found that the five response categories of an FV self-efficacy questionnaire were not used effectively, suggesting limitation in the response format. Similarly, item-response modeling among Hong Kong Chinese children identified a misfitting FSE item, differential item functioning across demographic groups, and inadequate coverage of the VSE trait range, despite acceptable-to-excellent internal consistency (17). Cultural adaptation among Brazilian adolescents also required linguistic and contextual modifications, including alignment with locally relevant dietary recommendations (18). Collectively, these findings indicate that a satisfactory Cronbach’s alpha does not, in itself, demonstrate factorial validity, appropriate item functioning, or adequate coverage of the underlying self-efficacy construct (17, 19). Therefore, the culturally adapted FSE and VSE scales require population-specific psychometric evaluation, including prespecified structural testing and transparent diagnostic investigation of model misfit; exploratory specifications should not be interpreted as validated measurement models.

To address this limitation, we adapted the existing FSE and VSE instruments to assess adolescents’ confidence in consuming fruits and vegetables across different behavioral situations and environmental contexts (10). The instrument conceptualizes SE as a behavior-specific construct and incorporates contextually relevant situations commonly experienced by adolescents, including meal routines, social influences, accessibility, and snack-related behaviors. This context-specific measurement matters because adolescents’ confidence in consuming fruits may differ from their confidence in consuming vegetables under varying environmental and social conditions (1, 8).

Culturally adapted measures may ultimately support research on adolescents’ fruit and vegetable self-efficacy and the evaluation of nutrition and health-promotion programs, but their broader use requires adequate population-specific psychometric evidence. Accordingly, this study aimed to culturally adapt the FSE and VSE scales and conduct an initial psychometric evaluation of the adapted item sets among secondary school adolescents in Selangor, Malaysia. Specifically, the study evaluated linguistic equivalence, content validity, face validity, pretest feasibility, item-level response distributions, floor and ceiling effects, internal consistency, composite reliability, standardized factor loadings and AVE, and the prespecified one-factor structure of each original 17-item scale. We evaluated the within-scale structures of FSE and VSE separately; therefore, we did not assess discriminant validity between the two constructs. Exploratory item-set sensitivity analyses examined the incremental contribution of one context-specific additional item to each scale, and *post hoc* diagnostic analyses were used solely to investigate possible sources of misfit in the prespecified VSE model. These exploratory analyses were hypothesis-generating and were not intended to establish a validated measurement model or readiness for broader application.

## 2 Materials and methods

This study was conducted in two sequential phases. Phase 1 involved translating and cross-culturally adapting the FSE and VSE scales for use among Malaysian adolescents, particularly in Selangor. This phase comprised forward and backward translation, cognitive debriefing, linguistic-equivalence assessment, evaluation of content and face validity, and pretesting. Phase 2 involved an initial psychometric evaluation in a separate school-based sample, including item-level performance, the prespecified factorial structure of each scale, internal consistency, composite reliability, and standardized factor loadings and AVE. The relevant subsections describe the participants, recruitment procedures, and findings for each phase.

### 2.1 Translation and cross-cultural adaptation of the fruit and vegetable self-efficacy instruments

We adapted the Fruit Self-Efficacy (FSE) and Vegetable Self-Efficacy (VSE) scales developed by Bannink and van der Bijl (10). Grounded in Bandura’s Social Cognitive Theory, these related but distinct scales assess adolescents’ confidence in consuming fruits and vegetables across a range of behavioral contexts and situational barriers. Each source scale consists of 17 items. The research team obtained permission from the instrument developers before commencing the adaptation. The multistage adaptation process comprised forward translation, linguistic equivalence assessment, reconciliation, cultural adaptation, expert content validation, face validity assessment, cognitive interviews, and pretesting, all conducted before the main psychometric evaluation (Supplementary Table S1).

#### 2.1.1 Forward and backward translation

The translation process followed established guidelines for the cross-cultural adaptation of self-report instruments (20). Four bilingual translators participated. All were native Malay speakers, proficient in English, familiar with health-related terminology, and experienced in developing health education materials. Two translators independently translated the original English instrument into Malay. Two other bilingual translators, who had not participated in the forward translation, subsequently and independently back-translated the Malay versions into English. The research team compared the forward and back-translated versions with the source instrument, discussed linguistic and conceptual discrepancies, and reconciled the translations by consensus. This process produced the preliminary Malay version designated FVSE_M0. The translators’ language backgrounds, relevant experience, specific roles, and independence are summarised in Supplementary Table S1f.

#### 2.1.2 Cognitive debriefing

To ensure linguistic clarity and cultural appropriateness, cognitive debriefing was conducted via a focus group discussion with five adolescents recruited through snowball sampling (20, 21). Recruitment was initiated through community contacts available to the research team, who referred potentially eligible adolescents. After parents or guardians provided consent, the focus group session was scheduled in consultation with them. The discussion was conducted in a private room at a community center to maintain confidentiality and minimize interruptions. Participants were aged 12–16 years and comprised three girls and two boys.

The researcher read aloud each item (original and translated) and reviewed them one at a time during the group discussion. Participants were asked whether the wording was understandable and appropriate and whether the item reflected the everyday experiences of Malaysian adolescents. They were also invited to explain the meaning of each item in their own words and identify any words, phrases, or situations that were difficult to understand or culturally unfamiliar. After reviewing all items, participants completed the questionnaire and provided general feedback on its length, perceived item repetition, ease of completion, and response burden. Feedback obtained during cognitive debriefing was used to refine several expressions and improve the linguistic clarity and cultural appropriateness of the preliminary Malay version.

#### 2.1.3 Linguistic-equivalence assessment

After reconciliation and cognitive debriefing, six experts in public health, nutrition, and educational psychology independently assessed the linguistic equivalence of the Malay version using a structured Google Forms checklist based on Sperber’s procedure (22). Each item was compared with its original English version to assess language comparability and interpretability. Both dimensions were rated on seven-point scales: from 1 (extremely comparable) to 7 (not comparable) and from 1 (extremely similar) to 7 (not similar), respectively, with lower mean scores indicating stronger equivalence.

Items exceeding the Sperber-based thresholds of 3.0 for language comparability or 2.5 for similarity of interpretability were considered potentially problematic. As a more conservative criterion, items with a mean score above 1.6 on either dimension or substantive expert comments were also reviewed. The research team used the ratings and comments to refine selected wording while preserving the intended conceptual meaning, after which the same panel reassessed the revised items. Supplementary Table S2 consolidates the original and revised wording, rationale for each modification, and item-level ratings before and after refinement.

### 2.2 Content validity assessment

Content validity was evaluated independently by nine experts purposively selected for their professional and research expertise in adolescent health, nutrition, psychology, public health, education, and questionnaire development. Item relevance and essentiality were assessed separately. For relevance, experts rated each item using a four-point scale. Ratings of 3 or 4 were classified as agreement that the item was relevant. The item-level Content Validity Index (I-CVI) was calculated by dividing the number of experts assigning a relevance rating of 3 or 4 by the total number of experts. The scale-level average Content Validity Index (S-CVI/Ave) was calculated as the arithmetic mean of the I-CVI values across the 17 source items. An I-CVI of at least 0.78 and an S-CVI/Ave of at least 0.90 were considered acceptable (23, 24).

Item essentiality was assessed separately from item relevance using a four-point response scale: 1 (*not essential at all*), 2 (*almost essential*), 3 (*essential*), and 4 (*very essential*). For the Content Validity Ratio (CVR) calculation, ratings of 1 or 2 were coded as 0 (*not essential*), whereas ratings of 3 or 4 were coded as 1 (*essential*). The item-level Content Validity Ratio (I-CVR) was calculated using Lawshe’s formula (25): I-CVR = $\left(N_{e}-N/2)/(N/2\right)$ , where $N_{e}$ represents the number of experts who classified an item as essential, and N represents the total number of experts. I-CVR values range from −1 to +1, with higher positive values indicating stronger agreement regarding item essentiality (25). For a panel of nine experts, an I-CVR of at least 0.78 was considered acceptable, corresponding to at least eight of the nine experts classifying an item as essential (26). The scale-level average Content Validity Ratio (S-CVR/Ave) was calculated as the arithmetic mean of the I-CVR values across the 17 source items and was interpreted descriptively, with values closer to 1.00 indicating stronger overall agreement regarding item essentiality.

Universal agreement was coded as 1 when all nine experts agreed and 0 when unanimity was not achieved; S-CVI/UA and S-CVR/UA represented the respective proportions of items achieving universal agreement (24). Assessments of item clarity and representativeness, together with written expert comments, were considered separately during the qualitative review and refinement of item wording; they were not used to calculate the reported I-CVI or I-CVR values. Panel members were also invited to identify behavioral situations that were not adequately represented in the source instruments and to propose contextually relevant additional or alternative items. Because all nine experts did not assess the proposed additional items independently as part of the original source-item set, they were classified as exploratory and excluded from the reported CVI and CVR calculations.

### 2.3 Face validity assessment

Face validity was assessed using complementary quantitative and qualitative methods involving 15 unique adolescents. Quantitative ratings provided standardized item and scale-level estimates of clarity and comprehensibility, whereas qualitative inquiry examined how intended respondents interpreted the wording and identified sources of difficulty. This approach was consistent with recommendations that response processes and face validity be assessed directly among the intended population (27–30). The two forms of evidence were considered together when assessing the clarity and comprehensibility of the adapted items and determining whether wording refinement was required.

We first assessed quantitative face validity among 10 adolescents aged 13–16 years, comprising six females and four males. We purposively selected three adolescents—one male and two females—and then used peer referrals over a two-week recruitment period, after which we enrolled 10 participants. Participants rated each item on a four-point scale for clarity and comprehensibility. We then calculated the Item-level Face Validity Index (I-FVI) and the Scale-Level Face Validity Index based on the average method (S-FVI/Ave). Values of at least 0.80 were considered acceptable (31, 32). The quantitative indices were based solely on ratings from these 10 participants (31, 32).

Items with an I-FVI below the prespecified threshold were reviewed and revised while preserving their intended meaning. Participants who initially assigned low clarity ratings reassessed the revised wording. We treated this procedure as a targeted reassessment rather than a complete repetition of the quantitative face-validity assessment. Therefore, the initial item-level findings were retained and reported transparently.

We subsequently assessed qualitative face validity in a separate session involving five adolescents. These participants were distinct from the 10 adolescents who completed the quantitative face-validity assessment. They reviewed the revised Malay items individually and were asked about each item’s clarity and comprehensibility, whether it contained difficult words or excessively long sentences, and whether its intended meaning was apparent. When an item was considered unclear or difficult to understand, participants were invited to explain the source of the difficulty and suggest alternative words or sentences that would be easier for adolescents to understand while preserving the intended meaning. We used their feedback to assess whether the concerns identified during cognitive debriefing had been adequately addressed and to guide further wording refinement where necessary.

Because no Chinese or Indian adolescents participated in the formal qualitative or quantitative face-validity assessments, an additional post-completion comprehension and clarity check was conducted with eight adolescents (four Indian and four Chinese) aged 13–16 years during the psychometric-evaluation phase. Immediately after returning the completed questionnaire, each adolescent was approached individually and asked whether they were willing to answer several questions about their experience of completing it. Participants were first asked whether the questionnaire was generally easy or difficult to answer. The researcher then referred sequentially to each FSE item by item number, followed by each VSE item, and asked whether they understood the item and found it easy or difficult to answer. The item wording was not read aloud by the researcher.

When a participant identified a difficulty, follow-up questions were used to determine which sentence, phrase, or word was unclear, whether the participant understood its meaning, and whether consulting the corresponding English wording helped clarify the item. Although all items were checked sequentially, the procedure did not include think-aloud responses, participant paraphrasing, or the detailed interpretive probing used in the formal cognitive-debriefing procedure. Because this additional post-completion comprehension and clarity check was not a prespecified formal cognitive-debriefing or face-validity procedure, the participants’ observations were reported separately and were not included in the formal face-validity sample or in the calculation of the I-FVI and S-FVI/Ave. The observations were treated as additional descriptive evidence of comprehensibility and administration feasibility among the eight adolescents assessed.

### 2.4 Pretesting of the adapted instruments

After completing the cultural adaptation and content and face validity assessments, the researchers pretested the final FSE and VSE instruments with 37 purposively recruited adolescents, consistent with recommended sample sizes for pretesting translated and culturally adapted questionnaires (20, 33). We purposively identified initial participants within the intended age range of 13–16 years and invited them to share the Google Forms survey link with adolescent peers. Recruitment therefore combined purposive sampling and peer referral. Through the peer-referral process, six participants outside the main target age range—one aged 10–12 years and five aged 17 years—also completed the pretest. We did not plan to include them as an extension of the main target population. Nevertheless, all six remained within the World Health Organization definition of adolescence, which encompasses individuals aged 10–19 years (34). We retained their responses because pretesting was limited to evaluating item comprehensibility, response-category use, questionnaire administration, and technical feasibility rather than confirming the factorial structure or establishing definitive psychometric properties.

The survey link remained open for 3 weeks, and participants completed the questionnaire online without direct researcher assistance. The pretesting sample comprised 25 girls (67.6%) and 12 boys (32.4%), aged 10 to 17 years. One participant (2.7%) was aged 10–12 years, 31 (83.8%) were within the main target age range of 13–16 years, and five (13.5%) were 17 years old. Within the target age range, three participants were 13 years old, one was 14 years old, 11 were 15 years old, and 16 were 16 years old. Most participants were Malay (*n* = 32, 86.5%), followed by Chinese (*n* = 4, 10.8%) and Bumiputera Sarawak (*n* = 1, 2.7%).

Pretesting examined item comprehensibility, questionnaire completion, item-level missing responses, use of the four response categories, online administration and data-capture procedures, and preliminary internal consistency. We calculated Cronbach’s alpha separately for the 17-item FSE and VSE scales and interpreted it only as a preliminary estimate within the pretesting sample. This phase was not designed to evaluate or confirm the factorial structure of either scale. Evidence obtained from participants outside the 13–16-year target range was not automatically generalized to the main secondary-school adolescent population. We then conducted the main psychometric evaluation using a separate sample of 128 adolescents aged 13–16 years.

### 2.5 Study design and participants of the psychometric evaluation

We conducted an initial psychometric evaluation as part of a larger school-based cross-sectional study examining the roles of health literacy and self-efficacy in adolescents’ fruit and vegetable intake in Selangor, Malaysia. Data were collected for psychometric evaluation in May 2025 during regular school hours using a structured, self-administered questionnaire.

Adolescents aged 13–16 years were recruited from a school-based sample. A single public secondary school in Selangor was purposively selected based on three criteria: (i) non-residential status, (ii) enrolment of more than 1,000 students, and (iii) representation of Malaysia’s major ethnic groups, Malay, Chinese, and Indian. Within the selected school, classes were stratified by grade level (Form 1, Form 2, Form 3, and Form 4), and one class from each grade level was randomly selected using a computer-based randomization procedure in Microsoft Excel. All eligible students in the selected classes were invited to participate. Students were eligible if they understood Malay or English and provided written assent and guardian consent. Students with medical conditions affecting dietary intake or who were absent during data collection were excluded. The final sample comprised 128 adolescents from 4 classes within the participating school.

A total of 144 students were assessed as eligible, and all were invited to participate. Seven students did not return parental or guardian consent forms, four did not provide adolescent assent, and five were absent on the scheduled data-collection day. We did not document the consent and assent status of the five absent students because they were not present during data collection. The remaining 128 adolescents had the required parental or guardian consent and adolescent assent, completed the assessment, and were included in the psychometric analyses. The overall response rate was 88.9% (128/144).

The pre-final FSE and VSE questionnaires administered during the psychometric-evaluation phase each comprised 17 source items and one exploratory context-specific additional item proposed during expert review. We included the additional items before the main data collection and did not introduce them in response to the psychometric findings. The original 17-item scales were the prespecified primary item sets, whereas the corresponding 18-item versions were examined only in exploratory item-set sensitivity analyses. The prespecified 17-item models, expanded 18-item models, and *post hoc* VSE diagnostic specification were all developed and evaluated using the same sample of 128 adolescents; no separate model-development or confirmation sample was available. We therefore treated findings from the expanded item sets and *post hoc* VSE analysis as exploratory and requiring independent evaluation. Detailed procedures are presented in the Psychometric Evaluation Procedures subsection.

During study planning, a minimum respondent-to-item ratio of 5:1 was used solely as a pragmatic recruitment heuristic. Based on the maximum of 18 indicators included in each separately estimated scale model, this approach yielded a minimum recruitment target of 90 participants. It was not used as a model-specific justification of sample-size adequacy for CFA. A total of 128 adolescents completed the psychometric evaluation. No *a priori* model-specific power analysis or Monte Carlo simulation was conducted. Because each prespecified CFA involved 17 ordered-categorical indicators with multiple response thresholds and was estimated using WLSMV, we could not determine the required sample size from a universal respondent-to-item ratio. Sample-size requirements depend on several interacting conditions, including factor loadings, category distributions, estimator performance, model complexity, residual parameters, and the extent of model misspecification (35–37). Accordingly, the achieved sample cannot be assumed to ensure adequate statistical power, precise and stable parameter estimates, or replicability. The CFA findings were therefore treated as preliminary and sample-specific. This limitation is particularly important for the *post hoc* VSE residual-covariance analysis, in which we identified and evaluated six residual covariances using the same sample.

The sample-size calculation was based on the nominal number of individual participants and did not account for the hierarchical sampling structure or a possible design effect from nesting students within classes (38). Because the sample comprised only four class-level clusters, the effective amount of independent information may have been lower than the nominal sample size of 128 suggests. With so few clusters, cluster-robust standard errors and a multilevel CFA model could not be estimated reliably (39, 40). This analytical constraint did not justify treating individual observations as independent because unmodelled within-cluster dependence may affect statistical inference in structural equation models (38). Consequently, the extent to which within-class dependence affected parameter estimates, standard errors, significance tests, and model-fit statistics could not be determined, and the inferential findings should be interpreted cautiously.

### 2.6 Measures and data collection for the main psychometric evaluation

Participants completed a structured, self-administered questionnaire during regular school hours, with minimal guidance from the research team. The questionnaire was in dual-language Malay and English and was presented in sequence as a single set of questionnaires. The questionnaire also collected sociodemographic characteristics, health literacy, and fruit and vegetable intake as part of the larger study. In the present psychometric report, these variables were used only to describe the sample; group comparisons and relationships with external variables were not included as evidence of psychometric validity. Completion required approximately 10–20 min, depending on class and age group.

#### 2.6.1 Sociodemographic characteristics

Participants were adolescents enrolled in the participating public secondary school in Selangor. They self-reported their sex, age, ethnicity, daily pocket money, and number of siblings. Ethnicity was categorized as Malay, Chinese, Indian, or other, as applicable. We assessed socioeconomic status using the Family Affluence Scale (FAS), which measures household material affluence based on indicators such as vehicle ownership, bedroom occupancy, family holidays, and access to household amenities. We categorized FAS scores into low, moderate, and high affluence groups (41).

#### 2.6.2 Fruit and vegetable intake

We assessed fruit and vegetable intake using a self-reported measure of daily serving frequency. Adequate fruit intake was defined as consuming at least two servings of fruit per day, whereas adequate vegetable intake was defined as consuming at least three servings of vegetables per day. We defined combined adequate fruit and vegetable intake as meeting both recommendations simultaneously. We adapted the instrument from a previously validated adolescent dietary assessment tool (42). To help respondents understand the term “serving,” we provided a visual serving-size guide alongside items referring to the number of fruit or vegetable servings. The guide illustrated examples of one serving using fruits and vegetables commonly consumed in Malaysia. The serving-size examples were based on the Malaysian Dietary Guidelines (43).

#### 2.6.3 Fruit and vegetable self-efficacy

Participants rated each item on a 4-point Likert-type scale ranging from 1 (“uncertain”) to 4 (“very certain”). Item responses were summed separately for descriptive FSE and VSE score summaries, with higher values indicating greater confidence in the corresponding consumption situations (10). Because an acceptable structural model was not established for VSE, its summed score was retained only for limited descriptive reporting and was not used for group comparisons or analyses of relationships with external variables.

### 2.7 Psychometric evaluation procedures

Because the FSE and VSE were existing instruments with prespecified structures, separate CFAs were conducted to evaluate their original one-factor models among adolescents in the target population (44, 45). We summarised the item sets separately by fruit and vegetable-specific content and source scoring structures, with no predefined subdomains. These analyses evaluated only the prespecified within-scale structure of each item set. Because the item sets were not evaluated simultaneously in a joint measurement model, we neither directly assessed nor established discriminant validity between FSE and VSE.

Items were treated as ordered-categorical indicators and estimated using the weighted least squares mean and variance-adjusted estimator (WLSMV) because of their four-category response format (37, 46). Model fit was evaluated using scaled χ^2^, χ^2^/df, CFI, TLI, RMSEA with its 90% confidence interval, and SRMR. Values of χ^2^/df < 3 indicated good fit and <5 acceptable fit, whereas CFI and TLI ≥ 0.90 and RMSEA and SRMR ≤0.08 indicated acceptable fit (45). These criteria were interpreted collectively as approximate guidelines rather than absolute decision rules (47).

The prespecified models were evaluated before any *post hoc* analysis. For the VSE diagnostic analysis, modification indices, expected parameter changes, standardized residuals, standardized factor loadings, explained item variance, and item content were considered collectively (44, 48). Residual covariances were introduced sequentially only when the paired items shared substantively related wording, contexts, or behavioral demands (49). Decisions regarding item retention or exclusion were informed by statistical and theoretical evidence, preservation of conceptual coverage, content-validity findings, cultural relevance, and feedback from experts and adolescents (20, 21). This *post hoc* analysis was used solely to investigate potential sources of VSE model misfit and was not treated as confirmatory evidence or as evidence of discriminant validity between FSE and VSE.

The primary evaluation used the original 17-item versions of FSE and VSE. Exploratory item-set sensitivity analyses compared these item sets with their corresponding expanded 18-item versions, each containing one context-specific additional item per scale (50). All item-set models were estimated as unmodified one-factor WLSMV models with uncorrelated residuals and were compared descriptively using the fit indices described above, standardized factor loadings, AVEs, and CRs. The prespecified 17-item VSE model used in this comparison differed from the *post hoc* diagnostic specification, which included six correlated residuals.

A second exploratory sensitivity analysis was conducted to determine whether the misfit of the initial unmodified 17-item VSE model could be attributed to an individual item. Each of the 17 source items was omitted in turn, and the resulting 16-item one-factor model was re-estimated using WLSMV with uncorrelated residuals. The leave-one-item-out models were compared descriptively using χ^2^/df, CFI, TLI, RMSEA with its 90% confidence interval, SRMR, and the remaining standardized factor loadings. Item retention was evaluated jointly based on the consistency and magnitude of changes across the fit indices, the standardized factor loadings, and preservation of construct coverage. Because the models examined in both sensitivity analyses contained different sets of observed indicators and were not strictly nested, formal χ^2^ difference testing was not conducted (51).

Standardized factor loadings and AVE were used to assess the convergence of the item indicators, with values ≥0.50 considered acceptable. Internal consistency was assessed using Cronbach’s alpha and McDonald’s omega, whereas CR was used to estimate composite reliability; coefficients ≥0.70 were considered acceptable (48). Corrected item–total correlations and reliability coefficients following item omission were also examined. AVE and CR were calculated from the standardized WLSMV loadings and corresponding residual variances. Test–retest reliability was not assessed because repeated measurements were unavailable, and measurement invariance was not examined because the sample size would have produced small and unequal subgroups.

### 2.8 Statistical analysis

Data were analyzed using IBM SPSS Statistics for Windows, version 30.0 (IBM Corp., Armonk, NY, United States), and JASP, version 0.98.1 (52). We checked all returned questionnaires item by item for completeness. No missing responses were identified; therefore, all 128 participants were included in the analyses, and no data imputation was required. Categorical variables were summarised using frequencies and percentages, whereas continuous variables were reported as means with standard deviations (SDs) or medians with interquartile ranges (IQRs), as appropriate. Participant characteristics were used only to describe the composition of the sample. We did not compare FSE or VSE scores across sociodemographic groups because we did not evaluate measurement invariance across groups.

We described item-level FSE and VSE responses using means and SDs. Response distributions were examined by calculating the number and proportion selecting each of the four categories for every item. Item-level floor and ceiling percentages were defined as the proportions selecting the lowest (1, “uncertain”) and highest (4, “very certain”) categories, respectively. A pronounced item-level floor or ceiling effect was operationally defined as at least 50% selecting the corresponding extreme category (53). For descriptive purposes, scale-level effects were assessed as the proportions attaining the theoretical minimum or maximum of each primary 17-item score, with proportions exceeding 15% considered indicative of floor or ceiling effects (54, 55). The descriptive VSE score was not interpreted as evidence of a coherent unidimensional construct and was not used for group comparisons or relationships with external variables.

We considered the potential non-independence of observations arising from student nesting within classes. However, participants were recruited from only four classes within one school, which was insufficient for reliable estimation of cluster-robust standard errors or a multilevel CFA model. The CFA analyses were therefore conducted at the individual level without adjustment for class-level clustering. Consequently, unmodelled within-class dependence may have affected the parameter estimates, standard errors, chi-square statistics, and model-fit indices. The findings were interpreted as preliminary in view of this sampling limitation.

### 2.9 Ethical considerations

This study formed part of a larger research project registered under the National Medical Research Register, Ministry of Health Malaysia (NMRR-24-03533-HIF). The Secretariat of Research Ethics at Universiti Kebangsaan Malaysia approved the study (JEP-2025-158). We obtained permission from school authorities before data collection and written informed consent from guardians, while adolescents provided written assent before participation. Participation was voluntary, and students could withdraw at any time without academic consequences. The study adhered to the principles of the Declaration of Helsinki. Clinical trial number: Not applicable.

## 3 Results

### 3.1 Cross-cultural adaptation

The cross-cultural adaptation involved forward translation, reconciliation, back translation, expert assessment of linguistic equivalence, cognitive debriefing with adolescents, and iterative item refinement. The source introductory stem, “I am certain that I can…,” was adapted as “Saya yakin saya boleh…,” *in the final Malay version, and* subsequently back-translated as “I am confident that I can…”. The Malay wording was selected because it conveyed confidence in performing the specified behavior and was considered natural and understandable for Malaysian adolescents. The response categories were translated as *tidak pasti*, *hampir pasti*, *pasti*, and *sangat pasti*. An item-level audit trail documenting the progression from the source version through translation, expert review, cognitive debriefing, content validity assessment, face validity assessment, pretesting, and finalization is presented in Supplementary Table S3a. Supplementary Table S3b presents the source English items, final Malay items, and back-translated English versions.

The cognitive debriefing and expert review findings informed several age-appropriate linguistic and contextual refinements. The term *snek/makanan ringan* was used to clarify “snack,” while “candy bar” was expressed using the more familiar terms *gula-gula* or *coklat*. The formal wording related to preparing food independently was simplified to “sediakan sendiri.” Practical examples were incorporated into FSE02 and VSE04 to clarify that preparation could involve washing, peeling, cutting, serving, or cooking. Examples of vegetables familiar to Malaysian adolescents that could be consumed as snacks were also added to VSE01. For social eating situations, “party” was contextualized as *majlis/kenduri*, which participants considered more familiar than *pesta* (Supplementary File S1a).

FSE12 required adaptation of the intake unit. A literal translation of “pieces of fruit” as *potongan buah* was interpreted by adolescents as referring only to cut pieces of fruit. Consequently, whole fruits, such as bananas or apples, were not consistently recognized as contributing to the required intake. Following feedback from the nutrition officer, “two or more pieces of fruit per day” was therefore expressed as *dua atau lebih hidangan buah sehari* (two or more servings of fruit per day). A pictorial serving guide was provided to illustrate fruit serving equivalents, including fruits commonly consumed whole and those usually consumed in slices or portions. This adaptation retained the intended daily intake of at least 2 servings of fruit while accommodating differences in fruit size and forms of consumption.

VSE17 also required contextual rather than literal translation. In the Malaysian context, a “hot meal” is generally understood as a cooked meal served warm or hot, most commonly associated with a main meal. A literal translation as *hidangan panas* could have been interpreted as referring only to food temperature and might not have clearly conveyed the intended eating situation to younger adolescents. The item was therefore adapted as eating vegetables during *makan utama*, with breakfast, lunch, and dinner provided as examples. This wording was intended to convey eating vegetables as part of a cooked main meal, rather than as a snack or an uncooked food. The change was documented as a contextual adaptation because the back-translated wording, “during main meals,” was not lexically identical to “during a hot meal” but was considered more functionally meaningful in the everyday eating context of Malaysian adolescents.

Participants further explained that vegetables were generally consumed as part of main meals, particularly lunch and dinner, whereas fruits were more commonly consumed as snacks or during leisure activities. Consequently, situations involving the consumption of vegetables as snacks were perceived as less consistent with their usual eating practices. Nevertheless, the original behavioral situations were retained to preserve the intended construct coverage and comparability of the FSE and VSE item sets. Two context-specific additional items were examined exploratorily during adaptation: purchasing fruit during leisure activities and consuming three or more servings of vegetables per day. Because these items were not included in the source scales, the primary item sets retained the original 17 FSE and 17 VSE items; findings for the expanded versions were treated as exploratory sensitivity analyses.

The six-expert linguistic-equivalence assessment yielded generally low mean scores, indicating generally favorable comparability between the source and translated versions. For the FSE scale, mean language-comparability scores ranged from 1.33 to 1.83, whereas mean similarity-of-interpretability scores ranged from 1.00 to 1.83. For the VSE scale, the corresponding scores ranged from 1.17 to 1.83 and from 1.33 to 2.00, respectively. No item exceeded the prespecified Sperber-based thresholds of 3.0 for language comparability or 2.5 for similarity of interpretability. Nevertheless, items with mean scores above the study-specific conservative criterion of 1.6 or substantive expert comments were reviewed and, where appropriate, refined. Following refinement, reassessed mean scores for the revised items ranged from 1.00 to 1.33 for language comparability and from 1.17 to 1.50 for similarity of interpretability, indicating improved linguistic comparability and similarity of meaning for the revised wording (Supplementary Table S2).

During pretesting, participants recommended administering the questionnaire in a paper-based format and providing clear instructions for completing it, with minimal guidance available for clarification. No further substantive changes to the retained item content were introduced after pretesting. Supplementary Tables S3a, S3b document the full progression of wording changes and the final questionnaire versions.

### 3.2 Content validity

All 17 source items in both scales met the prespecified item-level criteria for relevance and essentiality. For FSE, I-CVI values ranged from 0.89 to 1.00, while I-CVR values ranged from 0.78 to 1.00. The S-CVI/Ave of 0.99 exceeded the prespecified scale-level criterion of 0.90, and the S-CVR/Ave of 0.99 indicated strong overall expert agreement regarding item essentiality. FSE16 had the lowest values for both relevance and essentiality (I-CVI = 0.89; I-CVR = 0.78), with eight of the nine experts rating the item as relevant and essential. All other FSE source items received I-CVI and I-CVR values of 1.00 (Supplementary Table S4).

For VSE, I-CVI values ranged from 0.78 to 1.00, with an S-CVI/Ave of 0.95, while I-CVR values ranged from 0.78 to 1.00, with an S-CVR/Ave of 0.96. The S-CVI/Ave exceeded the prespecified criterion of 0.90, and the S-CVR/Ave indicated strong average expert agreement regarding item essentiality. VSE07, VSE10, and VSE11 received the lowest item-level values (I-CVI = 0.78; I-CVR = 0.78), but met the prespecified minimum criteria. All 17 source items were therefore retained. Expert comments informed targeted refinements to improve semantic clarity, cultural relevance, and age appropriateness (Supplementary Tables S4c, S4d). One additional context-specific item was proposed for each scale; because all nine experts did not assess these items independently as part of the original source-item set, they were classified as exploratory and excluded from the reported CVI and CVR calculations. Item-level content-validity findings are reported in Supplementary Table S4.

### 3.3 Face validity

Quantitative face validity was subsequently assessed among 10 adolescents using the pre-final 18-item versions, comprising the 17 source items and one exploratory additional item per scale. For FSE, clarity I-FVI values ranged from 0.70 to 1.00 and comprehensibility I-FVI values from 0.90 to 1.00, with S-FVI/Ave values of 0.97 for both domains. FSE-Add received I-FVI values of 1.00 for clarity and comprehensibility. For VSE, clarity and comprehensibility I-FVI values ranged from 0.80 to 1.00 and from 0.90 to 1.00, respectively, with corresponding S-FVI/Ave values of 0.95 and 0.96. VSE-Add received I-FVI values of 0.90 for both domains.

FSE02 (“…eat fruit if I have to prepare it myself”) obtained an initial clarity I-FVI of 0.70, below the prespecified threshold of 0.80, although its comprehensibility I-FVI was 0.90. Its wording was revised by adding concrete examples of simple fruit preparation, including washing, peeling, cutting, and serving. During targeted reassessment, the three adolescents who had initially assigned low clarity ratings changed their ratings to 3, 4, and 3, respectively. This reassessment determined only whether the specific difficulty identified by those respondents had been addressed; it was not a repeat face-validity assessment, and no revised I-FVI was calculated. The initial I-FVI of 0.70 was retained and reported. Given its content validity findings (I-CVI = 1.00; I-CVR = 1.00), the revised item was retained in the primary 17-item FSE set.

The qualitative face-validity assessment involving five adolescents indicated that most items were generally understood, although several items presented item-specific linguistic and contextual difficulties. Participants requested clarification of the wording in FSE02 and VSE04 regarding preparing fruit and vegetables, including examples such as washing, peeling, cutting, serving, and cooking. Questions were also raised about terms referring to servings or portions, main or hot meals, desserts, and social events such as “party” or “pesta.” Several vegetable-related situations required additional contextual clarification. In particular, some participants questioned the plausibility of eating vegetables as snacks or as substitutes for biscuits, crisps, or chocolate, and noted similarities among some snack-related items. The wording of VSE12 also prompted discussion because purchasing vegetables was viewed by some participants as a responsibility generally undertaken by parents rather than adolescents. This feedback informed the use of simpler wording, the removal of unfamiliar expressions, and the inclusion of familiar examples where appropriate, while preserving the intended meaning of the source items. Face validity and targeted reassessment results are presented in Supplementary Table S5, and the progression of item modifications is shown in Supplementary Table S3.

After completing the psychometric-evaluation questionnaire, we conducted an additional post-completion comprehension and clarity check with eight adolescents, comprising four Indian and four Chinese participants. Participants generally reported that the questionnaire was clear, comprehensible, and manageable. The only recurring difficulty concerned the Malay term “hidangan” (“serving”) (see Supplementary File S1b). When clarification was required, participants referred to the corresponding English wording or sought assistance from the researcher. As this additional check was not a prespecified formal cognitive-debriefing or face-validity procedure, the eight participants were reported separately and were not included in the formal cognitive-debriefing or face-validity samples or in the calculation of the I-FVI and S-FVI/Ave. Their observations provide additional descriptive evidence of questionnaire comprehensibility and administration feasibility but were not interpreted as demonstrating consistent interpretation or cross-group interpretive equivalence.

### 3.4 Pretesting of the adapted instruments

During pretesting, each pre-final instrument comprised 17 items from the respective source scale and one additional exploratory, context-specific item. A total of 42 adolescents accessed the online questionnaire, of whom 37 (88.1%) completed and submitted it. Among the 37 participants, one (2.7%) was aged 10–12 years, 31 (83.8%) were aged 13–16 years, and five (13.5%) were aged 17 years. Within the target range, three participants were aged 13 years, one was aged 14 years, 11 were aged 15 years, and 16 were aged 16 years.

No item-level missing responses were identified among the 37 completed questionnaires, and all four response categories were represented across the FSE and VSE item responses. Re-examination of the 31 participants aged 13–16 years similarly identified no item-level missing responses or restricted use of response categories. Participants generally found the adapted wording and examples comprehensible. The principal clarification required concerned the serving-size illustration and the interpretation of fruit and vegetable intake quantities. Participants indicated that the illustration should remain clear and legible, particularly in the printed questionnaire. No other major comprehension, administrative, or technical difficulties were identified.

Analyses of the 17-item source scales showed that FSE-17 scores ranged from 17 to 68 (*M* = 50.76, *SD* = 15.09), while VSE-17 scores also ranged from 17 to 68 (*M* = 39.00, *SD* = 15.82). For FSE-17, McDonald’s ω was 0.972 (95% CI: 0.948–0.983), and Cronbach’s α was 0.971 (95% CI: 0.947–0.983). For VSE-17, ω was 0.970 (95% CI: 0.952–0.982), and α was 0.969 (95% CI: 0.948–0.982). These coefficients were calculated from the complete pretesting sample of 37 participants and should be interpreted only as preliminary estimates. Separate reliability estimates were not calculated for the 31 participants aged 13–16 years because the modest subsample would not support sufficiently stable interpretation. Coefficients exceeding 0.95, along with participants’ comments about questionnaire length and item repetition, raised concerns about content redundancy. Feedback concerning vegetables consumed as snacks also indicated that the contextual relevance of these situations required further evaluation.

Because no major feasibility concerns were identified, all 17 source items in each scale were retained for the main psychometric evaluation. The two additional context-specific items were retained only for exploratory item-set sensitivity analyses and were not incorporated into the primary scales. Their development and pretesting are documented in Supplementary Table S3, while their exploratory psychometric evaluation is reported in Section 3.8 and Supplementary Table S9. The pretesting findings were interpreted only as evidence concerning comprehensibility, response-category use, administration, technical feasibility, and preliminary internal consistency; they were not considered confirmation of the factorial structure or definitive psychometric properties of either scale. Findings involving participants outside the 13–16-year range were not generalized to the main target population.

### 3.5 Psychometric evaluation participants’ demographics

Table 1 shows that 55.5% of participants were male and 44.5% were female. Their mean age was 14.36 years (SD = 1.06). Indian adolescents constituted the largest ethnic group (46.9%), followed by Malay (33.6%) and Chinese adolescents (16.4%); adolescents from other ethnic groups accounted for 3.1%. Approximately 39.8% reported high family affluence. Participants generally reported inadequate intake of fruits and vegetables. Mean daily intake was 2.20 fruit servings (SD = 1.25) and 2.02 vegetable servings (SD = 0.89). Only 7.0% met both recommendations, defined as consuming at least 2 fruit and 3 vegetable servings per day.

**Table 1:** Sociodemographic characteristics and fruit and vegetable intake among adolescents (*N* = 128).

Variable	*n*	%	Mean (SD)
Sex			
Male	71	55.5	
Female	57	44.5	
Ethnicity			
Malay	43	33.6	
Chinese	21	16.4	
Indian	60	46.9	
Others	4	3.1	
Age (years)			14.36 (1.06)
13	35	27.3	
14	33	25.8	
15	39	30.5	
16	21	16.4	
Family affluence scale (FAS)			
Low	27	21.1	
Moderate	50	39.1	
High	51	39.8	
Vegetable intake (servings/day)			2.02 (0.89)
Inadequate	105	82.0	
Adequate	23	18.0	
Fruit intake (servings/day)			2.20 (1.25)
Inadequate	91	71.1	
Adequate	37	28.9	
Combined fruit and vegetable intake (≥2 fruit and ≥3 vegetable servings/day)			
Inadequate	119	93.0	
Adequate	9	7.0	

Values are presented as frequency (*n*), percentage (%), or mean (standard deviation [SD]). Adequate fruit intake was defined as ≥2 servings/day and adequate vegetable intake as ≥3 servings/day. Combined adequate intake required participants to meet both recommendations simultaneously.

### 3.6 Item-level descriptive statistics

The questionnaire administered during the psychometric-evaluation phase contained 18 FSE items and 18 VSE items: the 17 items from each source instrument and one exploratory context-specific additional item per scale. Accordingly, item-level response distributions were summarised for all 18 administered items. In contrast, scale-score summaries, scale-level floor and ceiling effects, and reliability estimates were based on the primary 17-item FSE and VSE scales, excluding the exploratory additional items. The two item sets were analyzed separately, with no predefined subdomains. Higher item scores indicated greater confidence in performing the specified behavior (Table 2).

**Table 2:** Item-level descriptive statistics and standardized factor loadings from the prespecified 17-item FSE and VSE models.

Item code	Item wording	Standardized factor loading	Mean	Standard deviation
Fruit self-efficacy English-Malay version				
FSE01	…eat fruit as a snack.	0.832	3.344	0.917
FSE02	…eat fruit if I have to prepare it myself, such as by washing, peeling, cutting, and serving it	0.734	3.406	0.778
FSE03	…eat fruit during lunch.	0.812	3.203	0.991
FSE04	…eat fruit if my parents are not with me.	0.824	3.430	0.893
FSE05	…eat fruit as a snack instead of biscuits.	0.862	3.266	0.952
FSE06	…eat fruit during a meal at home.	0.710	3.227	0.974
FSE07	…eat fruit at breakfast.	0.805	3.031	1.136
FSE08	…eat fruit even if no one else is eating fruit.	0.841	3.367	0.963
FSE09	…eat fruit as a snack instead of sweets or chocolate (bar).	0.772	3.016	1.027
FSE10	…eat fruit when I am with my friends.	0.810	3.344	0.934
FSE11	…eat fruit as a snack instead of crisps.	0.784	3.000	1.072
FSE12	…eat 2 or more servings of fruit per day (see the serving information).	0.838	3.328	0.948
FSE13	…eat fruit at a social event or feast if it is available.	0.788	3.258	0.916
FSE14	…eat fruit as a dessert after a meal.	0.781	3.242	0.986
FSE15	…remember to bring fruit to school as a snack.	0.789	2.844	1.146
FSE16	…buy fruit if there is none at home.	0.647	2.523	1.157
FSE17	…ask a family member to buy fruit for me.	0.748	3.406	0.873
Vegetable self-efficacy English-Malay version				
VSE01	…eat vegetables as a snack.	0.858	2.289	1.028
VSE02	…eat vegetables even if no one else is eating them.	0.837	2.828	1.095
VSE03	…eat vegetables at lunchtime.	0.869	3.172	1.013
VSE04	…eat vegetables if I have to prepare them myself.	0.792	2.453	1.156
VSE05	…eat vegetables when my parents are not with me.	0.840	2.867	1.068
VSE06	…eat vegetables when I eat with my friends.	0.892	2.883	1.069
VSE07	…eat vegetables as a snack instead of biscuits.	0.884	2.203	1.166
VSE08	…eat vegetables at a party or family gathering if they are served.	0.859	2.695	1.140
VSE09	…eat vegetables as a snack instead of crisps.	0.854	2.125	1.115
VSE10	…eat vegetables as a snack instead of chocolate.	0.874	2.188	1.121
VSE11	…remember to bring vegetables to school as a snack.	0.694	2.461	1.196
VSE12	…buy vegetables if there are none at home.	0.696	2.289	1.211
VSE13	…ask a family member to cook vegetables for a main meal.	0.855	2.961	1.060
VSE14	…eat vegetables when I eat at home.	0.897	3.180	1.023
VSE15	…eat four or more spoonfuls of vegetables at each meal.	0.853	2.820	1.139
VSE16	…ask a family member to buy vegetables.	0.822	2.945	1.110
VSE17	…eat vegetables at my main meals (breakfast, lunch, and dinner).	0.894	3.164	1.063

Across the 18 items administered for each construct, endorsement of the lowest response category ranged from 1.6 to 25.8% for FSE and from 9.4 to 38.3% for VSE. Endorsement of the highest response category ranged from 28.1 to 63.3% for FSE and from 14.8 to 53.9% for VSE. No item demonstrated a pronounced floor effect, based on the operational criterion of at least 50% endorsement of the lowest response category. Pronounced item-level ceiling effects were observed for 12 of the 18 administered FSE items (66.7%; FSE01–FSE06, FSE08, FSE10, FSE12–FSE14, and FSE17) and three of the 18 administered VSE items (16.7%; VSE03, VSE14, and VSE17). Neither context-specific additional item demonstrated a pronounced ceiling effect. Thus, responses to a substantial proportion of the administered FSE items were concentrated in the highest response category, indicating restricted item-level response variability. These descriptive findings raise concerns regarding the ability of some FSE items to discriminate among adolescents with relatively high fruit self-efficacy; however, item discrimination and redundancy were not directly evaluated using item-response methods.

For the primary 17-item scales, the median FSE score was 57.00 (IQR = 17.25; M = 54.23, SD = 12.03), whereas the median VSE score was 46.00 (IQR = 21.00; M = 45.52, SD = 14.28). Observed scores ranged from 17 to 68 for both scales, covering their full possible ranges. These total scores are reported descriptively and, particularly for VSE, should not be interpreted as representing an established unidimensional construct.

At the scale-score level, one participant (0.8%) obtained the minimum possible FSE score, and 12 participants (9.4%) obtained the maximum possible FSE score. In VSE, 2 participants (1.6%) obtained the minimum possible score, and 8 participants (6.3%) obtained the maximum possible score. All proportions were below the prespecified 15% criterion, indicating no substantial scale-level floor or ceiling effects for either primary 17-item scale. Therefore, the concentration of responses in the highest category for individual FSE items did not produce a corresponding scale-level ceiling effect. Nevertheless, the absence of a scale-level ceiling effect does not exclude restricted response variability or potentially limited discrimination at the individual-item level. Complete item-level response distributions and scale-score findings are presented in Supplementary Table S6.

### 3.7 Confirmatory factor analysis and reliability

CFA was conducted separately for the original 17-item FSE and VSE scales (Table 3). The prespecified one-factor FSE model demonstrated acceptable global fit: scaled *χ*^2^(119) = 190.973, *p* < 0.001; *χ*^2^/df = 1.60; CFI = 0.974; TLI = 0.970; RMSEA = 0.069 (90% CI: 0.050–0.087); and SRMR = 0.061. These results provided initial, sample-specific support for the prespecified one-factor structure of the original 17-item FSE scale.

**Table 3:** Confirmatory factor analysis model-fit and composite reliability results for the prespecified 17-item fruit and vegetable self-efficacy models.

Parameter	Fruit self-efficacy	Vegetable self-efficacy
Items	17	17
Scaled *χ*^2^ (df)	190.973 (119)	396.605 (119)
*χ*^2^/df	1.60	3.33
CFI	0.974	0.952
TLI	0.970	0.945
RMSEA (90% CI)	0.069 (0.050–0.087)	0.136 (0.121–0.150)
SRMR	0.061	0.099
Standardized loading range	0.647–0.862	0.694–0.897
AVE	0.622	0.708
CR	0.965	0.976

FSE, Fruit self-efficacy; VSE, Vegetable self-efficacy; CFA, confirmatory factor analysis; CFI, Comparative fit index; TLI, Tucker–Lewis Index; RMSEA, root mean square error of approximation; CI, confidence interval; SRMR, standardized root mean square residual; AVE, average variance extracted; CR, composite reliability. Both original 17-item scales were specified as separate one-factor models without correlated residuals and estimated using the weighted least squares mean and variance-adjusted (WLSMV) estimator for ordered-categorical indicators (*N* = 128). Scaled *χ*^2^ values and robust fit indices are reported. AVE and CR were calculated from the standardized factor loadings. All standardized factor loadings were statistically significant at *p* < 0.001.

In contrast, the prespecified one-factor VSE model demonstrated substantial global misfit: scaled *χ*^2^(119) = 396.605, *p* < 0.001; *χ*^2^/df = 3.33; CFI = 0.952; TLI = 0.945; RMSEA = 0.136 (90% CI: 0.121–0.150); and SRMR = 0.099. Although the CFI and TLI exceeded 0.90, these incremental fit indices did not offset the substantial misfit indicated by the RMSEA and SRMR. Accordingly, the prespecified model did not support a sufficiently coherent unidimensional VSE construct, and the structural validity of the VSE scale was not established in this sample.

All standardized factor loadings in the original 17-item models were statistically significant (*p* < 0.001). FSE loadings ranged from 0.647 to 0.862, with corresponding *R*^2^ values ranging from 0.419 to 0.742. VSE loadings ranged from 0.694 to 0.897, with corresponding *R*^2^ values ranging from 0.481 to 0.805. AVE was 0.622 for FSE and 0.708 for VSE. These item-level findings describe indicator performance within the specified models; the favorable VSE loadings and AVE did not override the inadequate global fit of the prespecified one-factor model. Complete item-level CFA findings are presented in Supplementary Table S8.

Both original 17-item scales demonstrated high internal consistency and composite reliability (FSE: *α* = 0.943, 95% CI: 0.918–0.959; ω = 0.943, 95% CI: 0.919–0.959; CR = 0.965; VSE: *α* = 0.955, 95% CI: 0.941–0.966; ω = 0.955, 95% CI: 0.942–0.966; CR = 0.976). These coefficients provide evidence concerning score consistency but do not establish the structural validity or unidimensional interpretation of the VSE total score. Detailed item-level internal-consistency findings, including corrected item–total correlations and reliability coefficients following individual item omission, are presented in Supplementary Table S7.

### 3.8 Exploratory CFA diagnostics and item-set sensitivity analyses

Across the 17 leave-one-item-out VSE models, CFI ranged from 0.948 to 0.962, TLI from 0.940 to 0.956, RMSEA from 0.125 to 0.144, and SRMR from 0.082 to 0.104. Deletion of VSE16 produced the largest relative improvements in CFI, TLI, and RMSEA, whereas deletion of VSE10 produced the lowest SRMR. Neither deletion nor omission of any other individual item produced acceptable global fit. Thus, the misfit of the prespecified VSE model could not be attributed to a single item (Supplementary Table S9b).

A separate *post hoc* diagnostic analysis examined six sequential residual covariances among VSE items with overlapping wording or contexts involving family assistance, snack substitution, and routine home or main-meal consumption. The complete sequence is reported in Supplementary Table S9c. Relative to the prespecified model, the model containing all six correlated residuals showed a lower χ^2^/df (2.11), higher CFI (0.978) and TLI (0.974), and a lower SRMR (0.067). RMSEA also decreased but remained above the prespecified criterion (RMSEA = 0.094, 90% CI: 0.077–0.110).

Consequently, the specification containing six correlated residuals did not achieve acceptable global fit. Because the residual covariances were identified and evaluated using the same sample of 128 adolescents, the observed relative improvement may be sample-specific. This *post hoc* specification is reported solely as a hypothesis-generating diagnostic result requiring independent evaluation and does not constitute a validated measurement model. It does not alter the primary conclusion that the VSE scale’s structural validity was not established.

In a separate exploratory item-set sensitivity analysis, the original 17-item scales were compared descriptively with their corresponding expanded 18-item versions using unmodified one-factor models with uncorrelated residuals. The original 17-item scales were retained as the primary item sets because the context-specific additional items did not demonstrate clear incremental psychometric value. For FSE, inclusion of FSE-Add was associated with less favorable global model fit. For VSE, inclusion of VSE-Add produced only marginal changes and did not resolve the elevated RMSEA and SRMR of the unmodified model. Retaining the original VSE item set preserved fidelity to the source instrument but should not be interpreted as confirmation of its one-factor structure. Findings for both expanded 18-item versions remain exploratory and require evaluation in an independent sample.

## 4 Discussion

This study culturally adapted and conducted an initial psychometric evaluation of the Fruit Self-Efficacy (FSE) and Vegetable Self-Efficacy (VSE) item sets among 128 adolescents recruited from one public secondary school in Selangor, Malaysia. The adaptation process provided preliminary, sample-specific evidence concerning cultural relevance, content validity, face validity, and administration feasibility (20, 21), while the original 17-item versions remained the primary item sets in accordance with the source instruments (10). The structural findings were scale-specific. The prespecified FSE-17 model demonstrated acceptable global fit without *post hoc* correlated residuals, providing initial sample-specific support for its one-factor structure. In contrast, the prespecified VSE-17 model demonstrated substantial global misfit. The findings should therefore be interpreted as an initial psychometric evaluation rather than definitive validation of either item set.

The adaptation findings demonstrate that conceptual and functional equivalence require more than literal translation and should be evaluated through input from members of the target population (20–22, 33). The quantitative face-validity indices provided preliminary evidence that most items were considered clear and comprehensible by the ten adolescents assessed. Overall, the qualitative face-validity findings were broadly consistent with these ratings and also identified item-specific difficulties that numerical indices alone could not adequately capture (Supplementary Table S5). This complementary contribution aligns with methodological guidance recommending that researchers interpret structured ratings alongside direct qualitative input from target respondents when evaluating item comprehensibility and response processes (32, 55, 56).

Although most items were generally understood, the qualitative face-validity assessment identified uncertainties concerning terminology and contexts related to fruit and vegetable preparation, serving or portion sizes, main meals, desserts, social occasions, and vegetables consumed as snacks or as substitutes for energy-dense snack foods. Participants’ feedback informed the use of simpler wording and more familiar examples while preserving the intended meaning of the source items. For example, translating “hot meal” as “main meal” preserved the intended eating context rather than referring literally to food temperature. These qualitative findings contextualized and complemented the quantitative face-validity ratings by identifying item-specific interpretation difficulties that numerical indices alone might not fully capture. This complementary use of structured ratings and direct input from intended respondents is consistent with methodological recommendations for evaluating item clarity, comprehensibility, and response processes during questionnaire adaptation (20, 32, 56).

The targeted reassessment of FSE02 produced acceptable clarity ratings from all three adolescents who had initially identified difficulty with the item, providing preliminary evidence that the addition of concrete preparation examples improved the revised wording for those participants. The additional post-completion comprehension and clarity check involving four Indian and four Chinese adolescents was also broadly consistent with and complemented the formal face-validity findings: participants generally considered the questionnaire clear and manageable, although the Malay term “hidangan” (“serving”) occasionally required clarification. Together, these findings provide complementary descriptive evidence concerning the clarity and comprehensibility of the adapted questionnaire among the adolescents assessed. Nevertheless, the FSE02 reassessment was limited to the three initially low-rating respondents, while the additional post-completion check was not a prespecified formal cognitive-debriefing or face-validity procedure and did not generate formal item-level ratings. The findings therefore neither represent a revised face-validity estimate for the complete ten-participant group nor establish consistent interpretation or cross-group interpretive equivalence across the wider target population (20, 32, 56).

Population coverage across the adaptation stages nevertheless remained limited. Cognitive debriefing and the separate qualitative face-validity assessment involved the same five adolescents, rather than two independently recruited groups. The quantitative face-validity assessment involved 10 adolescents, and the targeted reassessment of FSE02 involved only 3 participants from that group. Consequently, the formal procedures did not provide sufficient representation of Selangor’s major ethnic and age groups. During administration of the psychometric-evaluation questionnaire, an additional post-completion comprehension check was conducted with eight adolescents, comprising four Indian and four Chinese participants. They generally reported that the questionnaire was clear and manageable. When they needed clarification, they could consult the corresponding English wording or ask a member of the research team for an explanation. The only recurring difficulty concerned the Malay term “hidangan” (“serving”), indicating that this term may still require particular attention in future cognitive testing. These observations provided supplementary descriptive information concerning comprehensibility and administration feasibility. However, this check was not a prespecified cognitive debriefing or face-validity procedure, did not produce formal ratings, and was not combined with the formal face-validity findings. It therefore neither established cross-group interpretive equivalence nor compensated for the limited population coverage of the formal adaptation procedures. Population coverage during pretesting was also uneven: 32 of the 37 participants (86.5%) were Malay, and 32 (86.5%) were aged 15–17 years. Broader cognitive interviewing and face-validity testing in independently recruited and purposively diverse ethnic and age groups are therefore required before consistent interpretation across the intended population can be inferred.

Pretesting provided preliminary evidence that the adapted items and administration procedures were feasible: all 37 submitted questionnaires were complete, and all four response categories were used. The original 17-item sets also produced very high preliminary internal-consistency estimates (FSE: *α* = 0.971, ω = 0.972; VSE: *α* = 0.969, ω = 0.970). These coefficients indicate strongly interrelated scores but do not establish optimal category functioning or unidimensionality; coefficients above 0.95 may also be influenced by repeated or overlapping item content (56, 57). Comments regarding questionnaire length and perceived repetition therefore warranted closer examination of item-level response patterns rather than immediate deletion of source items.

Although the prespecified FSE-17 model demonstrated acceptable global fit, the item-level distributions revealed an important limitation. Pronounced ceiling effects occurred in 12 of the 18 administered FSE items, compared with three of the 18 administered VSE items. The concentration of FSE responses in the highest category did not produce a substantial scale-level ceiling effect, but it may restrict item-level variability and reduce discrimination among adolescents with relatively high fruit self-efficacy. Similar behavioral situations may also indicate overlapping content and may contribute to high internal-consistency estimates. Acceptable global fit does not eliminate concerns about response concentration, redundancy, or discrimination because these are distinct measurement considerations (55, 56, 58). The FSE findings therefore provide initial support for its prespecified structure but not evidence of optimal item-level performance or readiness for routine use. Independent studies using item-response or Rasch-based methods could assess category functioning, item difficulty, information, and targeting across the self-efficacy continuum (16, 17, 19).

The CFA findings must also be considered in relation to sample size. The main evaluation included 128 participants for each 17-item model with four-category ordered indicators. Such models estimate multiple item thresholds and factor loadings in addition to residual and factor parameters. The respondent-to-item ratio used during planning represented only a minimum recruitment target; it did not demonstrate adequate statistical power or parameter stability. No model-specific power analysis or Monte Carlo simulation was conducted, and the achieved sample therefore cannot be claimed to provide stable estimates merely because it exceeded a simple participants-per-item rule (35, 36). This limitation is especially important for the *post hoc* residual-covariance analysis. All structural estimates and fit indices should consequently be regarded as sample-specific and interpreted cautiously.

The prespecified VSE-17 model did not provide adequate structural evidence. Although its CFI and TLI exceeded 0.90, the RMSEA of 0.136 and SRMR of 0.099 indicated substantial global misfit. Fit indices should be interpreted collectively, particularly for ordered-categorical models; comparatively favorable incremental indices do not override substantial misfit indicated by other indices (47, 59, 60). Likewise, statistically significant loadings, favorable AVE, and high Cronbach’s alpha, McDonald’s omega, and composite reliability describe standardized factor loadings and AVE or score consistency but do not compensate for inadequate global model fit (44, 61). Similar or overlapping items may also elevate these coefficients. Accordingly, they were not used to defend the VSE structure. The structural validity of VSE was not established, and its total score should not be interpreted as a validated measure of a single latent dimension at present.

The *post hoc* residual-covariance analysis did not alter that conclusion. A specification containing six correlated residuals showed lower *χ*^2^/df and SRMR and higher CFI and TLI than the prespecified VSE-17 model, but its RMSEA remained above the prespecified criterion (RMSEA = 0.094, 90% CI: 0.077–0.110). Correlated residuals can arise from shared wording, item order, similar contexts, or method-related variance, and their inclusion does not, in itself, validate the intended measurement structure (49, 62). Moreover, the residual covariances were identified and evaluated within the same sample of 128 participants, without an independent confirmation sample, increasing the possibility of capitalizing on sample-specific characteristics (63). The specification is therefore reported solely as a hypothesis-generating diagnostic result requiring independent cross-validation and does not constitute a validated measurement model (64, 65).

The sampling design introduced an additional limitation that could not be corrected analytically in the present dataset. Participants were recruited from only four classes within one purposively selected school, yet the CFA observations were analyzed at the individual level. With only four clusters, a reliable cluster-adjusted or multilevel model could not be estimated. This inability does not justify assuming that responses within classes were independent. Unmodelled within-class similarity may have affected standard errors, significance tests, and model-fit statistics, although its direction and magnitude cannot be determined from these data. The results should therefore be interpreted as conditional on this unaddressed dependence and replicated using a multisite design with enough schools or classes to support cluster-adjusted estimation (44, 51).

The observed local dependencies nevertheless generate testable hypotheses about the VSE item content. Some items assess confidence in eating vegetables, whereas others also depend on availability, purchasing autonomy, food preparation, meal routines, or assistance from family members. These distinctions may be relevant in Malaysia, where adolescent dietary behavior is shaped by household provision, school food environments, accessibility, and competing energy-dense options (66–68). Studies in other adolescent populations likewise identify parental influence, preparation, accessibility, and sensory characteristics as influences on fruit and vegetable intake (69–71). Items involving family assistance may partly invoke proxy efficacy rather than personal efficacy alone (8, 72). These explanations were not directly measured and should be treated as hypotheses for future cognitive interviewing and prespecified model testing, not as confirmed causes of the observed residual associations.

The exploratory item-set sensitivity analyses did not show sufficient incremental benefit from either context-specific additional item. Although both additional items loaded on their intended factors, the inclusion of FSE-Add was associated with less favorable global fit, whereas VSE-Add produced only marginal changes and did not resolve the elevated RMSEA and SRMR. A strong loading alone does not establish conceptual necessity or improved measurement performance; item decisions should also consider content coverage, redundancy, global fit, and independent replication (58, 73). Retaining the original 17-item sets preserved fidelity to the source instruments but should not be interpreted as confirmation of optimal item composition or structural validity. The additional items remain exploratory and require evaluation in an independent sample before they are considered for future inclusion.

Discriminant validity between FSE and VSE was not directly evaluated. The item sets were analyzed separately according to their intended fruit and vegetable-specific content and the scoring approach of the source instruments, but separate CFA models assess only within-scale structure and cannot establish that the corresponding latent constructs are empirically distinguishable (44, 51). Furthermore, the substantial misfit of the prespecified VSE model precluded a defensible conclusion about its distinction from FSE. Differences in item content, descriptive scores, or associations with other variables should therefore not be interpreted as evidence of discriminant validity. Future research should first establish an adequate VSE measurement structure and then examine FSE and VSE simultaneously in a prespecified joint measurement model using an independent sample.

Consistent with the unsupported VSE structure, group comparisons and relationships with external variables based on their total score were not retained as psychometric evidence. A summed-score analysis would assume a sufficiently coherent construct, which was not established. Group-difference claims were also not retained because measurement invariance across sex, age, and ethnicity was not examined; the comparison involving an “Other” ethnic group of four participants was especially unstable (74, 75). Relationships of FSE or VSE with health literacy or dietary intake were likewise not retained as validity evidence. Measurement invariance across sex, age, and ethnicity was not evaluated; therefore, score comparability across these groups was not established. Evidence based on relations with external variables was also not examined. These measurement properties should be evaluated in independent and adequately powered samples, and analyses involving the VSE total score should be undertaken only after an adequate structural model has been established.

The practical implications should remain proportionate to the available evidence. The FSE-17 findings are preliminary and do not establish readiness for routine use or broader group-level research because of the limited adaptation coverage, sample-size uncertainty, unaddressed class clustering, single-school recruitment, substantial item-level ceiling effects, and the absence of test–retest reliability, responsiveness, and item-response analyses. The VSE-17 total score should not be used as though it arose from a confirmed unidimensional structure. Neither item set should currently support individual screening, diagnostic classification, high-stakes decisions, or definitive intervention evaluation. Independent replication in larger, ethnically and developmentally diverse, multisite samples is needed before broader use is recommended. Future studies should evaluate temporal stability, responsiveness where longitudinal use is intended, item-response properties, measurement invariance, class or school clustering, and discriminant validity through joint measurement modeling (54, 55, 76).

## 5 Conclusion

This study culturally adapted and conducted an initial psychometric evaluation of the FSE and VSE scales among 128 adolescents recruited from one public secondary school in Selangor, Malaysia. The findings provided initial evidence regarding the cultural relevance, content validity, face validity, and internal consistency of the adapted item sets. However, the structural and item-level findings differed substantially between the two scales. The prespecified FSE-17 model demonstrated acceptable global fit without *post hoc* correlated residuals, providing initial sample-specific support for its one-factor structure. Nevertheless, pronounced ceiling effects across 12 of the 18 administered FSE items raise concerns regarding restricted item-level variability and the ability of these items to discriminate among adolescents with relatively high fruit self-efficacy. In addition, test–retest reliability, responsiveness, and item-response properties were not evaluated. The present findings therefore do not establish that the FSE scale is ready for routine use or broader group-level research.

In contrast, the prespecified VSE-17 model demonstrated substantial global misfit (RMSEA = 0.136; SRMR = 0.099) and failed to establish structural validity. Although the *post hoc* specification containing six correlated residuals showed relative improvement in several fit indices, its RMSEA remained above the prespecified criterion of 0.080 (RMSEA = 0.094). High factor loadings, AVE, internal consistency, and composite reliability did not compensate for the inadequate global fit. Moreover, because the residual covariances were identified and evaluated using the same modest sample without independent confirmation, the *post hoc* specification may be sample-specific. It should therefore be regarded only as a hypothesis-generating diagnostic result and does not constitute a validated measurement model. Consequently, the VSE total score should not presently be interpreted as a validated measure of a single latent dimension.

Discriminant validity between FSE and VSE was not established because the two item sets were analyzed separately rather than simultaneously within a joint measurement model. Accordingly, the separate CFA findings provide information about the within-scale structure of each item set but do not demonstrate that FSE and VSE represent empirically distinguishable latent constructs. Differences in item content, descriptive scores, or psychometric findings should therefore not be interpreted as evidence of discriminant validity.

The two context-specific additional items did not demonstrate sufficient incremental psychometric value and were therefore not incorporated into the primary 17-item sets. Retention of the original items preserved fidelity to the source instruments but should not be interpreted as definitive validation. Overall, the findings should be regarded as evidence from cultural adaptation and initial psychometric evaluation rather than confirmation that either scale is ready for broader application. Independent replication in larger, more diverse, and multisite adolescent samples is required. Further evaluation of FSE should include test–retest reliability, item-response properties, and, where longitudinal or intervention use is intended, responsiveness before routine or broader group-level use is recommended. Further evaluation of VSE should include item-level qualitative investigation and independently prespecified structural analyses before its total score is used as a unidimensional measure. Future studies should also evaluate FSE and VSE simultaneously in a prespecified joint measurement model to assess their discriminant validity, alongside measurement invariance and potential school-level clustering.

## Data Availability

The original contributions presented in the study are included in the article/Supplementary material, further inquiries can be directed to the corresponding author.
