# Supplementary material for: Cross-cultural adaptation and initial psychometric evaluation of the fruit and vegetable self-efficacy scales among secondary school adolescents in Selangor, Malaysia
Source: Front Public Health. 2026 Sep 29;14:1920784. doi: 10.3389/fpubh.2026.1920784 (PMC13658580; doi:10.3389/fpubh.2026.1920784)
Supplement: Supplementary file 1 [file Supplementary_file_1.docx]

**Supplementary files S1**

**S1a. Translation Validation:**

**Cognitive debriefing through Focus Group Discussion (FGD)**

Instrument	: Fruit and Vegetable Intake Self-Efficacy Questionnaire, 18-item, Malay version	(FVSE_M0.1)

Participant	: Adolescents, purposive sampling

Number of participants : 5 (3 girls, 2 boys)

Age	: 12-16

Date	: 23 November 2024

Objective: To explore participants' interpretations of the translated items and to assess their linguistic clarity, cultural relevance, contextual appropriateness for Malaysian adolescents, and perceived response burden.

**Procedure:**
Each translated item was read aloud and reviewed individually. After each item, participants were asked whether they did not understand any words or phrases, whether any wording was inappropriate, and whether the item was relevant to the context and everyday experiences of Malaysian adolescents. Participants were also invited to explain the meaning of each item in their own words and to suggest more suitable wording when necessary. After all items had been reviewed, participants completed the questionnaire and were asked about their overall experience, including the questionnaire's length, repetitive items, ease of completion, feelings while answering, and any difficulties encountered.

FGD participants expressed views similar to those reported for the Fruit Self-Efficacy (FSE) scale:

i. Participants perceived several items in the fruit and vegetable sections as repetitive because they assessed similar situations or behaviours using parallel wording (e.g., F1/V1, F5/V7, and F11/V9).
ii. Some participants found the complete questionnaire relatively lengthy and suggested it could be shortened.
iii. The repetition and similarity of several items across the two sections made the questionnaire feel monotonous and potentially tiring to complete.
iv. Participants explained that vegetables are generally consumed as part of main meals, particularly lunch and dinner, whereas fruits are more commonly consumed as snacks or during leisure activities. These culturally situated eating patterns may affect the perceived relevance and interpretation of certain self-efficacy items, particularly those concerning vegetables consumed as snacks.
v. Some participants experienced difficulty interpreting several translated terms, including terms referring to a social celebration ("party"), a serving or portion of food ("serving"), and preparing food ("preparation"). These terms were replaced with simpler, more familiar Malay expressions based on participants' explanations and suggestions, while preserving the original items' intended meaning.
vi. Following these wording refinements, participants generally understood the intended meaning of the items. The remaining feedback primarily focused on item repetition, questionnaire length, and the contextual relevance of specific fruit- and vegetable-eating situations.

**S1b. Additional Post-Completion Comprehension and Clarity Check**

**Instrument:**
Fruit and Vegetable Self-Efficacy Questionnaire, 18-item Malay version, with corresponding English wording available for reference (FVSE_M0.4)

**Participants:**
Adolescents who had completed the questionnaire during the main psychometric-evaluation phase

**Number of participants:**
Eight adolescents

**Participant composition:**

- Indian adolescents: four participants (two boys and two girls)
- Chinese adolescents: four participants (two boys and two girls)

**Age range:**
13–16 years

**Objective:**
To obtain supplementary descriptive feedback concerning the comprehensibility, clarity, and administration feasibility of the Malay version of the questionnaire among Indian and Chinese adolescents who were not adequately represented in the formal cognitive-debriefing and face-validity procedures.

**Nature and status of the procedure:**
This additional post-completion comprehension and clarity check was conducted after participants had completed the psychometric-evaluation questionnaire. It was designed to obtain supplementary descriptive feedback from Indian and Chinese adolescents who were not adequately represented in the formal cognitive-debriefing and face-validity procedures. It was not administered as a repeat formal cognitive-debriefing session or quantitative face-validity assessment.

**Procedure:**
After participants returned the completed questionnaire, the researcher approached each participant individually and asked for permission to ask several questions about its clarity and ease of completion. Participants were initially asked whether the questionnaire was generally easy or difficult to answer. The researcher subsequently referred to each FSE item in numerical order, followed by each VSE item, and asked whether the item was understandable and easy or difficult to answer. The researcher did not read the item wording aloud. When a participant reported difficulty, the researcher asked targeted follow-up questions to identify the relevant sentence, phrase, or word, determine whether the participant understood its meaning, and establish whether the corresponding English wording aided comprehension. The procedure constituted an additional item-by-item post-completion comprehension and clarity check; it did not include think-aloud techniques, participant paraphrasing, or the detailed probing of interpretation undertaken during formal cognitive debriefing.

**Guiding questions:**

1. Was the questionnaire generally clear and easy to complete?
2. Were there any words, phrases, or questions that were difficult to understand?
3. Which specific word, phrase, or item was unclear?
4. What did you think the item was asking?
5. Did you refer to the corresponding English wording when the Malay wording was unclear?
6. Did you request clarification from a member of the research team?
7. Are there any words or examples that could make the questionnaire easier to understand?

**Documentation and interpretation:**
We documented participants' comments descriptively as supplementary field observations. No formal clarity or comprehensibility ratings were collected, no I-FVI or S-FVI/Ave was calculated, and no formal qualitative coding or saturation assessment was undertaken. We did not combine findings from these eight participants with those from the cognitive-debriefing or face-validity samples.

**Summary of observations:**
Participants generally reported that the questionnaire was clear, comprehensible, and manageable. When they needed clarification, they could refer to the corresponding English wording or ask a member of the research team for help. The only recurring difficulty concerned the Malay term "hidangan" ("serving"). Apart from this terminology issue, no recurring difficulties in interpreting the questionnaire items were reported.

**Interpretive limitation:**
These findings provide additional descriptive evidence regarding the comprehensibility and administration feasibility of the Malay questionnaire among the eight Indian and Chinese adolescents assessed. However, because no formal item-level ratings, systematic cognitive-interview procedures, qualitative coding, or saturation assessment were undertaken, the findings should not be interpreted as establishing cross-group interpretive equivalence or as replacing formal evaluation in a larger and purposively diverse sample.
