# Supplementary material for: Cross-cultural adaptation and initial psychometric evaluation of the fruit and vegetable self-efficacy scales among secondary school adolescents in Selangor, Malaysia
Source: Front Public Health. 2026 Sep 29;14:1920784. doi: 10.3389/fpubh.2026.1920784 (PMC13658580; doi:10.3389/fpubh.2026.1920784)
Supplement: Supplementary file 2 [file Table_1.docx]

Overview of Supplementary Materials

| **Supporting table** | **Information reported** |
| --- | --- |
| **Supplementary Table S1** | Stage-level summary of participants, sample sizes, recruitment or selection procedures, methods, principal findings, and resulting decisions across translation, adaptation, pretesting, and the main psychometric evaluation |
| **Supplementary Table S2** | Linguistic-equivalence assessment, including expert ratings of language comparability and similarity of interpretability, expert comments, wording revisions, and reassessment findings |
| **Supplementary Table S3** | Item-level audit trail documenting the progression from the source wording through translation, cognitive debriefing, expert review, content and face validity, pretesting, and finalization |
| **Supplementary Table S4** | Content-validity findings, including I-CVI, I-CVR, S-CVI/Ave, S-CVR/Ave, expert feedback, and resulting actions |
| **Supplementary Table S5** | Face-validity findings, including I-FVI, S-FVI/Ave, participant-level ratings, targeted reassessment of FSE02, and resulting decisions |
| **Supplementary Table S6** | Item- and scale-level response distributions and floor and ceiling effects for the FSE and VSE scales |
| **Supplementary Table S7** | Item-level internal-consistency findings, including corrected item–total correlations and reliability coefficients following individual item omission |
| **Supplementary Table S8** | Complete item-level CFA findings, including standardized factor loadings, standard errors, confidence intervals, statistical significance, and explained item variance. |
| **Supplementary Table S9** | Exploratory item-set sensitivity analyses comparing the original 17-item and expanded 18-item FSE and VSE versions, including model-fit indices, factor-loading ranges, AVE, and composite reliability. |
| **Supplementary File S1** | **S1a:** Cognitive-debriefing documentation, including participant characteristics, objectives, procedures, guiding questions, linguistic and contextual difficulties, participant feedback, wording revisions, and resulting decisions. **S1b:** Additional Post-Completion Comprehension and Clarity Check involving eight Indian and Chinese adolescents, including participant characteristics, procedures, clarification strategies, descriptive observations, and interpretive limitations. Participants generally considered the questionnaire clear and manageable, although the Malay term “hidangan” occasionally required clarification. |

Supplementary Table S1

Table S1a. Translation, cross-cultural adaptation, and initial psychometric evaluation of the Malay version of the fruit and vegetable self-efficacy (FVSE) scales

| **Phase** | **Step** | **Description** | **Participants** | **Method / Output** |
| --- | --- | --- | --- | --- |
| **Translation** | Forward translation | Translation of the original FVSE questionnaire into Malay | 2 bilingual translators | First draft of Malay version |
|  | Backward translation | Translation of the Malay version back into English | 2 bilingual translators | Reviewed the Malay version (FVSE_M0) |
| **Translation and cross-cultural adaptation** | Cognitive debriefing (adolescents) | Exploration of item interpretation, cultural relevance, contextual appropriateness, and perceived response burden | 5 adolescents | Focus group discussion (FGD) /FVSE_M0.1 |
|  | Experts  review | Assessment of language comparability and similarity of interpretability | 6 experts | Structured checklist (Google Form) |
| **Content validity (CVI)** | Content-validity assessment | Assessment of item relevance and representativeness for the intended constructs and target population | 9 experts | CVI tools-via google form/  FVSE_M0.2 |
| **Face validity** | Quantitative face-validity assessment | Quantitative assessment of item clarity and comprehensibility | 10 adolescents | FVI tools via Google Form |
|  | Qualitative - face-validity assessment | Evaluation of item comprehensibility, clarity of meaning, appropriateness of terminology, and sentence structure | 5 adolescents | FGD- feedback /  FVSE_M0.3 |
| **Pretesting** | Initial pilot testing | Assessment of missing or 'don’t know’ responses, item comprehension, participant comments, item repetition, and questionnaire feasibility | 37 adolescents | Questionnaire via Google Form |
| **Psychometric evaluation** | Main psychometric evaluation | Evaluation of the measurement properties of the adapted FSE and VSE scales – CFA, internal consistency, construct reliability. | 128 adolescents | Face-to-face, self-administered questionnaire  FVSE_M0.4 |
| **Finalisation** | Final instrument | Finalized Malay version of the FVSE questionnaire | — | FVSE_M1 |

Note. The adaptation followed a multistage process comprising forward and backward translation, cognitive debriefing, expert assessments, quantitative and qualitative face-validity assessments, pilot testing, and an initial psychometric evaluation. The stages are presented in the order in which they were undertaken.

Table S1b. Participants and recruitment or selection procedures across the adaptation, pretesting, and initial psychometric-evaluation stages

| Phase | Participant | Recruitment/selection procedure |
| --- | --- | --- |
| Forward translation | Two bilingual translators | Purposively selected based on bilingual proficiency; both independently translated the source instrument from English into Malay. |
| Backward translation | Two different bilingual translators | Purposively selected based on bilingual proficiency and had not participated in the forward translation; both independently back-translated the Malay version into English. |
| Cognitive debriefing | Five adolescents aged 12–16 years (three girls and two boys) | Recruited through initial community contacts followed by snowball referral. Parental or guardian consent and adolescent assent were obtained before participation. |
| Linguistic-equivalence assessment | Six experts from public health, nutrition, instrument development, education, and health-behavior research | Purposively selected based on relevant professional expertise and experience in language-equivalence and questionnaire assessment. |
| Content-validity assessment | Nine experts in adolescent health, nutrition, psychology, public health, education, and questionnaire development | Purposively selected based on their professional and research expertise relevant to the constructs, target population, and instrument development. |
| Quantitative face-validity assessment | Ten adolescents aged 13–16 years (six girls and four boys) | Recruitment began with three purposively identified participants (one boy and two girls), followed by peer referral until the required sample was obtained. The three participants who initially assigned low clarity ratings to FSE02 were invited for targeted reassessment. |
| Qualitative face-validity assessment | The same five adolescents who participated in cognitive debriefing | The five adolescents were invited to participate in a separate qualitative face-validity session; none of them participated in quantitative face validity. |
| Pretesting | Thirty-seven adolescents aged 10–17 years (25 girls and 12 boys); 31 of them were 13- 16 years | We purposively recruited initial eligible participants from the target population and invited them to share the Google Forms survey link with eligible peers. Recruitment therefore combined purposive and peer-referral approaches. |
| Main psychometric evaluation | 128 adolescents aged 13–16 years from four classes in one public secondary school | We purposively selected one school using predefined criteria. Classes were stratified by grade level, and one class from each of Forms 1–4 was randomly selected using computer-generated randomization. All eligible students in the selected classes were invited to participate. |

Table 1c. Recruitment and participation across the cultural-adaptation and psychometric-evaluation phases

| Phase | Identified | Invited | Accepted/ Consented/ assented | Decline/  Did not complete* | Completed | Completation rate, % |
| --- | --- | --- | --- | --- | --- | --- |
| Forward translation | 2 | 2 | 2 | 0 | 2 | 100% |
| Backward translation | 2 | 2 | 2 | 0 | 2 | 100% |
| Cognitive debriefing | 6  (3 girls, 3 boys) | 6  (3 girls, 3 boys) | 6  (3 girls, 3 boys) | 1  (boy) | 5  (3 girls, 2 boys) | 83% |
| Linguistic-equivalence assessment | 10 | 10 | 9 | 3* | 6 | 67% |
| Content-validity assessment | 10 | 9 | 9 | 0 | 9 | 100% |
| Quantitative face-validity assessment | 10 | 10 | 10 | 0 | 10 | 100% |
| Targeted reassessment of FSE02 | 3 | 3 | 3 | 0 | 3 | 100% |
| Qualitative face-validity assessment | 5 | 5 | 5 | 0 | 5 | 100% |
| Pretesting | 42 | 42 | 42 | 5* | 37 | 88% |
| Additional Post-Completion Comprehension and Clarity Check | 10 | 10 | 10 | 2 | 8 | 80% |

**Notes:** Completion rate was calculated as the number who completed the relevant study phase divided by the number who accepted, consented, or assented to participate, multiplied by 100. “Accepted” applies to translators and experts, whereas parental or guardian consent and adolescent assent apply to adolescent participants. The five adolescents who participated in the qualitative face-validity assessment were distinct from the 10 adolescents who completed the quantitative face-validity assessment. The targeted reassessment of FSE02 involved three participants from the quantitative face-validity group and was not a separately recruited sample.

Table 1d. Recruitment and participant flow across the cultural adaptation and psychometric evaluation phases.

| **Recruitment indicator** | **Main psychometric evaluation** |
| --- | --- |
| Assessed as eligible | 144 |
| Invited | 144 |
| Provided parental/guardian consent and adolescent assent | 133 |
| Did not provide the required consent or assent | 16   - Parental/guardian consent not returned (n = 7); - Adolescent assent not provided (n = 4); - Absent on the data-collection day, with consent/assent status not documented (n = 5) |
| Completed and included in the analysis | 128 |
| Response rate, % | 88.9 |

Note. Response rate was calculated as the number of participants who completed the assessment divided by the number of eligible students invited, multiplied by 100. Completion rate was calculated as the number who completed the assessment divided by the number who had provided parental or guardian consent and adolescent assent, multiplied by 100.

Table 1e. Expert panel involved in language-equivalence and content-validity assessments

| Expert role | Linguistic-equivalence assessment  (n = 6) | Content validation  (n = 9) | Years of professional experience (range) |
| --- | --- | --- | --- |
| Health education officers | 2 | 3 | 10-20 |
| Nutrition officer | 1 | 1 | 10 |
| Instrument development specialists | 1 | 2 | 10-20 |
| Educator | 1 | 1 | 5 |
| Academic researchers (public health/health behavior) | 1 | 2 | 15-20 |

Supplementary Table S1f. Qualifications, language backgrounds, experience, roles, and independence of the translators

| Translator | Translation role | Native language | Language proficiency | Experience | Independence |
| --- | --- | --- | --- | --- | --- |
| FT1 | Forward translation: English to Malay | Malay | Proficient in Malay and English | >10 years, experienced in developing health-education materials and familiar with health-related terminology | Translated independently |
| FT2 | Forward translation: English to Malay | Malay | Proficient in Malay and English | >10 years, experienced in developing health-education materials and familiar with health-related terminology | Translated independently |
| BT1 | Backward translation: Malay to English | Malay | Proficient in Malay and English | >10 years, experienced in developing health-education materials and familiar with health-related terminology | Not involved in forward translation; translated independently |
| BT2 | Backward translation: Malay to English | Malay | Proficient in Malay and English | >10 years, experienced in developing health-education materials and familiar with health-related terminology | Not involved in forward translation; translated independently |
