# Supplementary material for: Cross-cultural adaptation and initial psychometric evaluation of the fruit and vegetable self-efficacy scales among secondary school adolescents in Selangor, Malaysia
Source: Front Public Health. 2026 Sep 29;14:1920784. doi: 10.3389/fpubh.2026.1920784 (PMC13658580; doi:10.3389/fpubh.2026.1920784)
Supplement: Supplementary file 3 [file Table_2.DOCX]

**Supplementary Table S2**

Table S2a. Characteristics of the expert panel involved in the linguistic-equivalence assessment

| Expert role | Linguistic-equivalence assessment  (n = 6) | Years of professional experience (range) |
| --- | --- | --- |
| Health education officers | 2 | 10-20 |
| Nutrition officer | 1 | 10 |
| Instrument development specialists | 1 | 10-20 |
| Educator | 1 | 5 |
| Academic researchers (public health/health behavior) | 1 | 15-20 |

Table S2b. Item-level language-comparability and similarity-of-interpretability ratings for the initial Malay FSE and VSE translations

A. Language equivalence

| Very equivalent |  |  | Moderately equivalent |  |  | Not at all equivalent |
| --- | --- | --- | --- | --- | --- | --- |
| 1 | 2 | 3 | 4 | 5 | 6 | 7 |

B. Equivalence of meaning

| Very similar |  |  | Moderately similar |  |  | Not at all similar |
| --- | --- | --- | --- | --- | --- | --- |
| 1 | 2 | 3 | 4 | 5 | 6 | 7 |

**Fruit Self-efficacy (FSE)**

| a)      Language equivalence | | | | | | | | | b) Equivalence of meaning | | | | | | | | |
| --- | --- | --- | --- | --- | --- | --- | --- | --- | --- | --- | --- | --- | --- | --- | --- | --- | --- |
|  | **P1** | **P2** | **P3** | **P4** | **P5** | **P6** | **SUM** | **AVG. SUM** |  | **P1** | **P2** | **P3** | **P4** | **P5** | **P6** | SUM | AVG. SUM |
| FSE1 | 1 | 1 | 2 | 2 | 1 | 1 | 8 | 1.33 | FSE1 | 1 | 2 | 2 | 1 | 1 | 1 | 8 | 1.33 |
| FSE2 | 1 | 1 | 2 | 2 | 1 | 1 | 8 | 1.33 | FSE2 | 1 | 1 | 1 | 1 | 3 | 1 | 8 | 1.33 |
| FSE3 | 1 | 1 | 2 | 2 | 1 | 1 | 8 | 1.33 | FSE3 | 1 | 2 | 2 | 1 | 2 | 2 | 10 | 1.67 |
| FSE4 | 1 | 1 | 1 | 2 | 3 | 1 | 9 | 1.50 | FSE4 | 1 | 2 | 1 | 1 | 1 | 1 | 7 | 1.17 |
| FSE5 | 2 | 1 | 1 | 2 | 3 | 2 | 11 | 1.83 | FSE5 | 1 | 2 | 1 | 1 | 1 | 1 | 7 | 1.17 |
| FSE6 | 2 | 1 | 1 | 2 | 1 | 2 | 9 | 1.50 | FSE6 | 1 | 2 | 2 | 1 | 3 | 2 | 11 | 1.83 |
| FSE7 | 2 | 1 | 1 | 2 | 1 | 2 | 9 | 1.50 | FSE7 | 1 | 1 | 3 | 1 | 2 | 1 | 9 | 1.50 |
| FSE8 | 2 | 1 | 1 | 2 | 2 | 2 | 10 | 1.67 | FSE8 | 1 | 1 | 2 | 1 | 1 | 1 | 7 | 1.17 |
| FSE9 | 1 | 1 | 2 | 2 | 2 | 1 | 9 | 1.50 | FSE9 | 1 | 1 | 2 | 1 | 1 | 1 | 7 | 1.17 |
| FSE10 | 1 | 1 | 2 | 2 | 2 | 1 | 9 | 1.50 | FSE10 | 1 | 1 | 3 | 1 | 1 | 1 | 8 | 1.33 |
| FSE11 | 1 | 1 | 2 | 2 | 2 | 1 | 9 | 1.50 | FSE11 | 1 | 2 | 2 | 1 | 1 | 1 | 8 | 1.33 |
| FSE12 | 1 | 1 | 2 | 2 | 3 | 1 | 10 | 1.67 | FSE12 | 1 | 2 | 2 | 1 | 1 | 2 | 9 | 1.50 |
| FSE13 | 2 | 1 | 2 | 2 | 2 | 2 | 11 | 1.83 | FSE13 | 1 | 2 | 1 | 1 | 3 | 1 | 9 | 1.50 |
| FSE14 | 1 | 1 | 1 | 2 | 3 | 1 | 9 | 1.50 | FSE14 | 1 | 1 | 3 | 1 | 2 | 1 | 9 | 1.50 |
| FSE15 | 1 | 1 | 2 | 2 | 1 | 1 | 8 | 1.33 | FSE15 | 1 | 1 | 1 | 1 | 1 | 2 | 7 | 1.17 |
| FSE16 | 2 | 1 | 2 | 2 | 1 | 1 | 9 | 1.50 | FSE16 | 1 | 1 | 1 | 1 | 1 | 1 | 6 | 1.00 |
| FSE17 | 2 | 1 | 1 | 2 | 2 | 1 | 9 | 1.50 | FSE17 | 1 | 1 | 2 | 1 | 1 | 1 | 7 | 1.17 |

**Vegetables Self-Efficacy (VSE)**

| a)      Language equivalence | | | | | | | | | b) Equivalence of meaning | | | | | | | |  |
| --- | --- | --- | --- | --- | --- | --- | --- | --- | --- | --- | --- | --- | --- | --- | --- | --- | --- |
| Vegetable | P1 | P2 | P3 | P4 | P5 | P6 | SUM | AVG. SUM |  | P1 | P2 | P3 | P4 | P5 | P6 | SUM | AVG. SUM |
| VSE1 | 1 | 2 | 2 | 1 | 1 | 1 | 8 | 1.33 | VSE1 | 2 | 2 | 2 | 2 | 2 | 2 | 12 | 2.00 |
| VSE2 | 1 | 1 | 1 | 1 | 3 | 1 | 8 | 1.33 | VSE2 | 2 | 2 | 2 | 2 | 1 | 1 | 10 | 1.67 |
| VSE3 | 1 | 2 | 2 | 1 | 2 | 2 | 10 | 1.67 | VSE3 | 2 | 2 | 1 | 2 | 2 | 3 | 12 | 2.00 |
| VSE4 | 1 | 2 | 1 | 1 | 1 | 1 | 7 | 1.17 | VSE4 | 1 | 2 | 1 | 1 | 2 | 1 | 8 | 1.33 |
| VSE5 | 1 | 2 | 1 | 1 | 1 | 1 | 7 | 1.17 | VSE5 | 1 | 2 | 1 | 1 | 2 | 1 | 8 | 1.33 |
| VSE6 | 1 | 2 | 2 | 1 | 3 | 2 | 11 | 1.83 | VSE6 | 1 | 2 | 1 | 1 | 2 | 2 | 9 | 1.50 |
| VSE7 | 1 | 1 | 3 | 1 | 2 | 1 | 9 | 1.50 | VSE7 | 1 | 2 | 2 | 1 | 1 | 3 | 10 | 1.67 |
| VSE8 | 1 | 1 | 2 | 1 | 1 | 1 | 7 | 1.17 | VSE8 | 1 | 2 | 2 | 1 | 1 | 2 | 9 | 1.50 |
| VSE9 | 1 | 1 | 2 | 1 | 1 | 1 | 7 | 1.17 | VSE9 | 2 | 2 | 2 | 2 | 1 | 2 | 11 | 1.83 |
| VSE10 | 1 | 1 | 3 | 1 | 1 | 1 | 8 | 1.33 | VSE10 | 2 | 2 | 1 | 2 | 1 | 3 | 11 | 1.83 |
| VSE11 | 1 | 2 | 2 | 1 | 1 | 1 | 8 | 1.33 | VSE11 | 2 | 2 | 2 | 2 | 2 | 2 | 12 | 2.00 |
| VSE12 | 1 | 2 | 2 | 1 | 1 | 2 | 9 | 1.50 | VSE12 | 2 | 2 | 2 | 2 | 2 | 2 | 12 | 2.00 |
| VSE13 | 1 | 2 | 1 | 1 | 3 | 1 | 9 | 1.50 | VSE13 | 2 | 2 | 2 | 1 | 2 | 1 | 10 | 1.67 |
| VSE14 | 1 | 1 | 3 | 1 | 2 | 1 | 9 | 1.50 | VSE14 | 1 | 2 | 2 | 2 | 1 | 3 | 11 | 1.83 |
| VSE15 | 1 | 1 | 1 | 1 | 1 | 2 | 7 | 1.17 | VSE15 | 2 | 2 | 2 | 2 | 1 | 1 | 10 | 1.67 |
| VSE16 | 1 | 1 | 2 | 1 | 1 | 1 | 7 | 1.17 | VSE16 | 1 | 2 | 1 | 1 | 1 | 2 | 8 | 1.33 |
| VSE17 | 1 | 2 | 3 | 1 | 1 | 1 | 9 | 1.50 | VSE17 | 1 | 2 | 1 | 2 | 2 | 3 | 11 | 1.83 |

Note: Ratings were made on seven-point scales, ranging from 1 (extremely comparable/similar) to 7 (not comparable/similar). Lower scores indicated stronger linguistic and semantic equivalence. Mean language-comparability scores >3.0 or mean similarity-of-interpretability scores >2.5 indicated that an item required further review. Items with mean scores >1.6 or substantive expert comments were additionally reviewed using a more conservative study-specific procedure.

Table S2c. Reassessment of revised FSE and VSE items following linguistic-equivalence review

| a)      Language equivalence | | | | | | | | | b) Equivalence of meaning | | | | | | | | |
| --- | --- | --- | --- | --- | --- | --- | --- | --- | --- | --- | --- | --- | --- | --- | --- | --- | --- |
|  | **P1** | **P2** | **P3** | **P4** | **P5** | **P6** | **SUM** | **AVG. SUM** |  | **P1** | **P2** | **P3** | **P4** | **P5** | **P6** | SUM | AVG. SUM |
| FSE5 | 1 | 1 | 1 | 2 | 1 | 2 | 8 | 1.33 | FSE3 | 1 | 1 | 2 | 1 | 1 | 2 | 8 | 1.33 |
| FSE12 | 1 | 1 | 1 | 1 | 1 | 1 | 6 | 1.00 | FSE6 | 1 | 1 | 2 | 1 | 1 | 1 | 7 | 1.17 |
| FSE13 | 2 | 1 | 1 | 2 | 1 | 1 | 8 | 1.33 | VSE1 | 2 | 1 | 1 | 1 | 1 | 2 | 8 | 1.33 |
| VSE3 | 1 | 1 | 2 | 1 | 1 | 2 | 8 | 1.33 | VSE2 | 1 | 1 | 1 | 2 | 1 | 1 | 7 | 1.17 |
| VSE6 | 1 | 1 | 1 | 1 | 2 | 1 | 7 | 1.17 | VSE3 | 2 | 1 | 1 | 1 | 2 | 1 | 8 | 1.33 |
|  |  |  |  |  |  |  |  |  | VSE7 | 1 | 2 | 1 | 1 | 1 | 2 | 8 | 1.33 |
|  |  |  |  |  |  |  |  |  | VSE9 | 2 | 1 | 2 | 1 | 1 | 1 | 8 | 1.33 |
|  |  |  |  |  |  |  |  |  | VSE10 | 1 | 2 | 1 | 2 | 1 | 1 | 8 | 1.33 |
|  |  |  |  |  |  |  |  |  | VSE11 | 1 | 1 | 2 | 1 | 2 | 1 | 8 | 1.33 |
|  |  |  |  |  |  |  |  |  | VSE12 | 1 | 1 | 1 | 2 | 1 | 2 | 8 | 1.33 |
|  |  |  |  |  |  |  |  |  | VSE13 | 1 | 1 | 1 | 1 | 2 | 1 | 7 | 1.17 |
|  |  |  |  |  |  |  |  |  | VSE14 | 1 | 2 | 2 | 2 | 1 | 1 | 9 | 1.50 |
|  |  |  |  |  |  |  |  |  | VSE15 | 2 | 1 | 2 | 1 | 1 | 1 | 8 | 1.33 |
|  |  |  |  |  |  |  |  |  | VSE17 | 1 | 1 | 1 | 2 | 2 | 1 | 8 | 1.17 |

References

Sperber AD. Translation and validation of study instruments for cross-cultural research. Gastroenterology. 2004;126(Suppl 1):S124–S128. doi:10.1053/j.gastro.2003.10.016. [PubMed](https://pubmed.ncbi.nlm.nih.gov/14978648/?utm_source=chatgpt.com)
