# Supplementary material for: Cross-cultural adaptation and initial psychometric evaluation of the fruit and vegetable self-efficacy scales among secondary school adolescents in Selangor, Malaysia
Source: Front Public Health. 2026 Sep 29;14:1920784. doi: 10.3389/fpubh.2026.1920784 (PMC13658580; doi:10.3389/fpubh.2026.1920784)
Supplement: Supplementary file 4 [file Table_3.docx]

Supplementary Table 3

Supplementary Table S3a. Item-level audit trail of translation and cross-cultural adaptation of the FSE and VSE scales

| **Item** | **Original** | Translation (FVSE_M0) | FVSE_M0.1 (After cognitive debriefing and linguistic-equivalence assessment”) | FVSE_M0.2 (After Content validity) | FVSE_M0.3 (After face validity) | FVSE_M0.4 (After pretesting) | FVSE_M1 |
| --- | --- | --- | --- | --- | --- | --- | --- |
|  | *Answer choices : (1) Uncertain (2) Almost certain (3) Certain (4) Very certain* | Pilihan jawapan :  (1) Tidak pasti (2) Hampir pasti (3) Pasti (4) Sangat pasti | Pilihan jawapan :  (1) Tidak pasti (2) Hampir pasti (3) Pasti (4) Sangat pasti | Pilihan jawapan :  (1) Tidak pasti (2) Hampir pasti (3) Pasti (4) Sangat pasti | Pilihan jawapan :  (1) Tidak pasti (2) Hampir pasti (3) Pasti (4) Sangat pasti | Participants recommended administering the questionnaire in paper-based format and providing clear instructions and giving an explanation on how to complete it. | |
|  | **I am certain that I can...** | Saya pasti saya boleh...... | Saya pasti saya boleh...... | Saya yakin saya boleh... |  | Pilihan jawapan :  (1) Tidak pasti (2) Hampir pasti (3) Pasti (4) Sangat pasti | Pilihan jawapan :  (1) Tidak pasti (2) Hampir pasti (3) Pasti (4) Sangat pasti |
| FSE01 | eat fruit as a snack | … makan buat sebagai makanan ringan | …makan buah sebagai snek/ makanan ringan | …makan buah sebagai snek/ makanan ringan | Saya yakin saya boleh makan buah sebagai makanan ringan/snek | Saya yakin saya boleh makan buah sebagai makanan ringan/snek | Saya yakin saya boleh makan buah sebagai makanan ringan/snek |
| FSE02 | eat fruit if I have to prepare it myself | … makan buah kalau saya menyediakannya sendiri | … makan buah kalau saya perlu menyediakannya sendiri | ...makan buah jika saya perlu sediakan sendiri | Saya yakin saya boleh makan buah jika saya perlu sediakan sendiri seperti membasuh, mengupas, memotong dan menghidang | Saya yakin saya boleh makan buah jika saya perlu sediakan sendiri seperti membasuh, mengupas, memotong dan menghidang | Saya yakin saya boleh makan buah jika saya perlu sediakan sendiri seperti membasuh, mengupas, memotong dan menghidang |
| FSE03 | eat fruit during lunch | … makan buah ~~semasa~~ makan tengahari | … makan buah ketika makan tengah hari | ...makan buah ketika makan tengah hari | Saya yakin saya boleh makan buah ketika makan tengah hari | Saya yakin saya boleh makan buah ketika makan tengah hari | Saya yakin saya boleh makan buah ketika makan tengah hari |
| FSE04 | eat fruit if my parents are not there | … makan buah kalau ibubapa saya tiada bersama | ...makan buah walaupun tanpa ibu bapa saya tidak ada bersama-sama saya | ....makan buah jika ibu bapa tidak ~~ada~~ bersama saya | Saya yakin saya boleh makan buah jika ibu bapa tidak bersama-sama saya | Saya yakin saya boleh makan buah jika ibu bapa tidak bersama-sama saya | Saya yakin saya boleh makan buah jika ibu bapa tidak bersama-sama saya |
| FSE05 | eat fruit as a snack instead of a biscuit | … makan buah sebagai makanan ringan dan bukannya biskut | … makan buah sebagai snek/ makanan ringan menggantikan biskut | ...makan buah sebagai snek/ makanan ringan menggantikan biskut | Saya yakin saya boleh makan buah sebagai makanan ringan/ snek menggantikan biskut | Saya yakin saya boleh makan buah sebagai makanan ringan/ snek menggantikan biskut | Saya yakin saya boleh makan buah sebagai makanan ringan/ snek menggantikan biskut |
| FSE06 | eat fruit during a meal at home | … makan buah semasa makan-makan di rumah | … makan buah semasa makan di rumah | ...makan buah ketika makan di rumah | Saya yakin saya boleh makan buah ketika makan di rumah | Saya yakin saya boleh makan buah ketika makan di rumah | Saya yakin saya boleh makan buah ketika makan di rumah |
| FSE07 | eat fruit during breakfast | … makan buah semasa sarapan | … makan buah semasa sarapan | ...makan buah ketika sarapan | Saya yakin saya boleh makan buah ketika sarapan | Saya yakin saya boleh makan buah ketika sarapan | Saya yakin saya boleh makan buah ketika sarapan |
| FSE08 | eat fruit even if no one else is eating fruit | … makan buah walau tiada sesiapa yang makan buah | … makan buah walaupun tiada sesiapa makan buah | ...makan buah walaupun tiada orang lain makan buah | Saya yakin saya boleh makan buah walaupun tiada orang lain makan buah | Saya yakin saya boleh makan buah walaupun tiada orang lain makan buah | Saya yakin saya boleh makan buah walaupun tiada orang lain makan buah |
| FSE09 | eat fruit as a snack instead of a candy bar | … makan buah sebagai makanan ringan dan bukannya gula-gula | … makan buah sebagai snek/ makanan ringan dan bukannya gula-gula/ coklat | ...makan buah sebagai snek/ makanan ringan menggantikan coklat | Saya yakin saya boleh makan buah sebagai snek/ makanan ringan menggantikan gua-gula/ coklat | Saya yakin saya boleh makan buah sebagai snek/makanan ringan menggantikan gua-gula/ coklat | Saya yakin saya boleh makan buah sebagai snek/makanan ringan menggantikan gula-gula/ coklat |
| FSE10 | eat fruit when eating together with my friends | … makan buah bila makan bersama dengan kawan-kawan saya | … makan buah bila makan bersama-sama dengan kawan-kawan saya | ...makan buah dengan kawan-kawan | Saya yakin saya boleh makan buah dengan kawan-kawan | Saya yakin saya boleh makan buah ketika bersama kawan-kawan | Saya yakin saya boleh makan buah ketika bersama kawan-kawan |
| FSE11 | eat fruit as a snack instead of crisps | … makan buah sebagai makanan ringan dan bukannya kerepek | … makan buah sebagai makanan snek/ makanan ringan dan bukannya kerepek | ...makan buah sebagai snek / makanan ringan menggantikan kerepek | Saya yakin saya boleh makan buah sebagai snek / makanan ringan menggantikan kerepek | Saya yakin saya bolehmakan buah sebagai snek/ makanan ringan menggantikan kerepek | Saya yakin saya boleh makan buah sebagai snek/ makanan ringan menggantikan kerepek |
| FSE12 | eat two or more pieces of fruit per day | … makan dua potongan atau lebih buah sehari | … makan dua potong~~an~~ atau lebih buah sehari | ...makan buah dua atau lebih hidangan sehari | Saya yakin saya boleh makan buah dua atau lebih hidangan sehari | Saya yakin saya boleh makan buah dua atau lebih hidangan sehari (rujuk maklumat berkaitan hidangan) | Saya yakin saya boleh makan buah dua atau lebih hidangan sehari (rujuk maklumat berkaitan hidangan) |
| FSE13 | eat fruit in social situations, such as at a party, if it is available | … makan buah dalam majlis sosial, seperti di majlis keramaian/ parti, kalau ianya ada | … makan buah dalam majlis sosial, seperti di majlis keramaian/ parti/ pesta, kalau ada | ...makan buah dimajlis / kenduri jika ada | Saya yakin saya boleh makan buah dimajlis/ kenduri jika ada | Saya yakin saya boleh makan buah dimajlis/ kenduri jika ada | Saya yakin saya boleh makan buah di majlis/ kenduri jika ada |
| FSE14 | eat fruit as dessert after a hot meal | … makan buah sebagai pencuci mulut selepas masakan panas | … makan buah sebagai pencuci mulut (dessert) selepas masakan panas | ...makan buah sebagai pencuci mulut selepas makan | Saya yakin saya boleh makan buah sebagai pencuci mulut selepas makan | Saya yakin saya boleh makan buah sebagai pencuci mulut selepas makan | Saya yakin saya boleh makan buah sebagai pencuci mulut selepas makan |
| FSE15 | remember to take fruit to school as a snack | … ingat untuk membawa buah kesekolah sebagai makanan ringan | … ingat untuk membawa buah ke sekolah sebagai snek /makanan ringan | ... ingat untuk bawa buah ke sekolah sebagai snek | Saya yakin saya boleh ingat bawa buah ke sekolah sebagai snek | Saya yakin saya boleh ingat bawa buah ke sekolah sebagai snek | Saya yakin saya boleh ingat bawa buah ke sekolah sebagai snek |
| FSE16 | buy fruit if there is none in the house | … beli buah kalau ianya tiada dirumah | … beli buah kalau ianya tiada dirumah | ...beli buah jika tiada di rumah | Saya yakin saya boleh beli buah jika tiada buah di rumah | Saya yakin saya boleh beli buah jika tiada buah di rumah | Saya yakin saya boleh beli buah jika tiada buah di rumah |
| FSE17 | ask someone in my family to buy fruit | … minta seseorang dalam keluarga saya membeli buah | … minta seseorang dalam keluarga saya membeli buah | ...minta ahli keluarga membelikan buah | Saya yakin saya boleh minta ahli keluarga membelikan buah | Saya yakin saya boleh minta ahli keluarga belikan buah | Saya yakin saya boleh minta ahli keluarga belikan buah |
| FSE_ADD | Suggestion: additional questions during expert reviewing and content validity need to be considered by the research team | | …beli buah semasa berjalan-jalan /beriadah (.*..buy fruit when I am out or doing activities.* | …beli buah semasa berjalan-jalan /beriadah (.*..buy fruit when I am out or doing activities.* | Saya yakin saya boleh beli buah semasa berjalan-jalan beriadah | Saya yakin saya boleh beli buah semasa berjalan-jalan beriadah | Retained only for exploratory item set sensitivity; not included in the primary FSE-17/VSE-17 scale  Saya yakin saya boleh beli buah semasa berjalan-jalan beriadah (I am confident I can *buy fruit when I am out or doing activities.* |
|  |  | |  |  |  |  |  |
|  | *Answer choices : (1) Uncertain (2) Almost certain (3) Certain (4) Very certain* | Pilihan jawapan :  (1) Tidak pasti (2) Hampir pasti (3) Pasti (4) Sangat pasti | Pilihan jawapan :  (1) Tidak pasti (2) Hampir pasti (3) Pasti (4) Sangat pasti | Pilihan jawapan :  (1) Tidak pasti (2) Hampir pasti (3) Pasti (4) Sangat pasti | Pilihan jawapan :  (1) Tidak pasti (2) Hampir pasti (3) Pasti (4) Sangat pasti | -Perlu dikendalikan menggunakan borang soal selidik (fizikal)  - Arahan menjawab soalan perlu jelas  - Minimal guidence | |
|  | I am certain that I can... | Saya pasti saya boleh...... | Saya pasti saya boleh...... | Saya yakin saya boleh... |  | Pilihan jawapan :  (1) Tidak pasti (2) Hampir pasti (3) Pasti (4) Sangat pasti | Pilihan jawapan :  (1) Tidak pasti (2) Hampir pasti (3) Pasti (4) Sangat pasti |
| VSE01 | ...eat vegetables as a snack | … makan sayur sebagai makanan ringan | … makan sayur sebagai snek/ makanan ringan | ...makan sayur sebagai snek/ makanan ringan | Saya yakin saya boleh makan sayur sebagai snek/ makanan ringan (contoh timun, tomato ceri, daun bayam goreng rangup, cendawan inoki goreng rangup, lobak merah) | Saya yakin saya boleh makan sayur sebagai snek/ makanan ringan (contoh timun, tomato ceri, daun bayam goreng rangup, cendawan inoki goreng rangup, lobak merah) | Saya yakin saya boleh makan sayur sebagai snek/ makanan ringan (contoh timun, tomato ceri, daun bayam goreng rangup, cendawan inoki goreng rangup, lobak merah) |
| VSE02 | ...eat vegetables even if no one else is eating vegetables | … makan sayur walau tiada sesiapa yang makan sayur | … makan sayur walaupun tiada sesiapa ~~yang~~ makan buah | ...makan sayur walaupun tiada sesiapa makan sayur | Saya yakin saya boleh makan sayur walaupun tiada sesiapa makan sayur | Saya yakin saya boleh makan sayur walaupun tiada sesiapa makan sayur | Saya yakin saya boleh makan sayur walaupun tiada sesiapa makan sayur |
| VSE03 | ...eat vegetables during lunch | … makan sayur semasa makan tengah hari | … makan buah ketika makan tengah hari | ...makan sayur semasa makan tengah hari | Saya yakin saya boleh makan sayur semasa makan tengah hari | Saya yakin saya boleh makan sayur ketika makan tengah hari | Saya yakin saya boleh makan sayur ketika makan tengah hari |
| VSE04 | ...eat vegetables if I have to prepare them myself | … makan sayur kalau saya menyediakannya sendiri | … makan sayur kalau saya perlu menyediakannya sendiri | ...makan sayur jika saya perlu sediakannya sendiri seperti memasak sayur, membuat salad sayur, membasuh dan memotong ulam | Saya yakin saya boleh makan sayur jika saya perlu sediakan sendiri, seperti membasuh, memotong, memasak sayur. | Saya yakin saya boleh makan sayur jika saya perlu sediakannya sendiri seperti membasuh memotong, memasak sayur | Saya yakin saya boleh makan sayur jika saya perlu sediakannya sendiri seperti membasuh memotong, memasak sayur |
| VSE05 | ...eat vegetables if my parents are not there | … makan sayur kalau ibubapa saya tiada | ..makan sayur walaupun tanpa ibu bapa saya tidak ada bersama-sama saya | ...makan sayur walaupun ibu bapa tidak ada bersama dengan saya. | Saya yakin saya boleh makan sayur jika ibu bapa tidak bersama-sama saya | Saya yakin saya boleh makan sayur jika ibu bapa tidak bersama-sama saya | Saya yakin saya boleh makan sayur jika ibu bapa tidak bersama-sama saya |
| VSE06 | ...eat vegetables when eating together with my friends | … makan sayur bila makan bersama dengan kawan-kawan saya | … makan sayur bila makan bersama-sama dengan kawan-kawan saya | ...makan sayur apabila makan bersama kawan-kawan | Saya yakin saya boleh makan sayur ketika makan bersama kawan-kawan | Saya yakin saya boleh makan sayur ketika makan bersama kawan-kawan | Saya yakin saya boleh makan sayur ketika makan bersama kawan-kawan |
| VSE07 | ...eat vegetables as a snack instead of a biscuit | … makan sayur sebagai makanan ringan dan bukannya biskut | makan buah sebagai snek/ makanan ringan dan bukannya biskut | ... makan sayur sebagai snek bukan biskut | Saya yakin saya boleh makan sayur sebagai snek bukan biskut | Saya yakin saya boleh makan sayur sebagai snek bukan biskut | Saya yakin saya boleh makan sayur sebagai snek bukan biskut |
| VSE08 | ..eat vegetables in social situations, such as at a party, if available | … makan sayur dalam keadaan bersosial, seperti di majlis keramaian, kalau ianya ada | … makan sayur dalam majlis sosial, seperti di majlis keramaian/ parti/ pesta, kalau ~~ianya~~ ada | ... makan sayur dimajlis keraian / kenduri jika disediakan | Saya yakin saya boleh makan sayur dimajlis keraian / kenduri jika disediakan | Saya yakin saya boleh makan sayur dimajlis keraian / kenduri jika disediakan | Saya yakin saya boleh makan sayur di majlis keraian/ kenduri jika disediakan |
| VSE09 | ...eat vegetables as a snack instead of crisps | … makan sayur sebagai makanan ringan dan bukannya kerepek | Makansayur sebagai snek/ makanan ringan dan bukannya kerepek | ... makan sayur sebagai snek/ makanan ringan menggantikan kerepek | Saya yakin saya boleh makan sayur sebagai snek/ makanan ringan menggantikan kerepek | Saya yakin saya boleh makan sayur sebagai snek/ makanan ringan menggantikan kerepek | Saya yakin saya boleh makan sayur sebagai snek/ makanan ringan menggantikan kerepek |
| VSE10 | ...eat vegetables as a snack instead of a candy bar | …makan sayur sebagai makanan ringan dan bukannya gula-gula | makan sayur sebagai snek/ makanan ringan dan bukannya gula-gula/ coklat | ...makan sayur sebagai snek/makanan ringan menggantikan coklat | Saya yakin saya boleh makan sayur sebagai snek/makanan ringan menggantikan coklat | Saya yakin saya boleh makan sayur sebagai snek/makanan ringan menggantikan coklat | Saya yakin saya boleh makan sayur sebagai snek/makanan ringan menggantikan coklat |
| VSE11 | ...remember to take vegetables to school as a snack | …ingat untuk membawa sayur ke sekolah sebagai makanan ringan | … ingat untuk membawa sayur ke sekolah sebagai snek /makanan ringan | ...ingat untuk bawa sayur ke sekolah sebagai snek/ makanan ringan | Saya yakin saya boleh ingat untuk bawa sayur ke sekolah sebagai snek/ makanan ringan | Saya yakin saya boleh ingat untuk bawa sayur ke sekolah sebagai snek/ makanan ringan | Saya yakin saya boleh ingat untuk bawa sayur ke sekolah sebagai snek/ makanan ringan |
| VSE12 | ...buy vegetables if there are none in the house | … beli sayur kalau ianya tiada di rumah | … beli sayur kalau tiada dirumah | ... beli sayur jika tiada sayur di rumah | Saya yakin sayaboleh beli sayur jika tiada sayur di rumah | Saya yakin sayaboleh beli sayur jika tiada sayur di rumah | Saya yakin saya boleh beli sayur jika tiada sayur di rumah |
| VSE13 | ...ask someone in my family to prepare vegetables for a hot meal | ...minta ahli keluarga saya masak sayur untuk makan panas-panas | ... minta ahli keluarga masak sayur untuk hidangan utama | ... minta ahli keluarga masak sayur untuk hidangan utama | Saya yakin saya boleh minta ahli keluarga masak sayur untuk hidangan utama | Saya yakin saya boleh minta ahli keluarga masak sayur untuk hidangan utama | Saya yakin saya boleh minta ahli keluarga masak sayur untuk hidangan utama |
| VSE14 | ...eat vegetables during a meal at home | ...makan sayur semasa makan di rumah | ..makan sayur ketika makan di rumah | ... makan sayur ketika makan di rumah | Saya yakin saya boleh makan sayur ketika makan di rumah | Saya yakin saya boleh makan sayur ketika makan di rumah | Saya yakin saya boleh makan sayur ketika makan di rumah |
| VSE15 | ...eat four or more serving spoons of vegetables per day | ...makan sayur empat atau lebih sudu setiap hari | ...makan sayur empat atau lebih sudu setiap hari | ... makan sayur empat atau lebih sudu bagi setiap hidangan (kali makan) | Saya yakin saya boleh makan empat atau lebih sudu sayur setiap hidangan (kali makan) | Saya yakin saya boleh makan empat atau lebih sudu sayur setiap hidangan (kali makan) | Saya yakin saya boleh makan empat atau lebih sudu sayur setiap hidangan (kali makan) |
| VSE16 | ...ask someone in my family to buy vegetables | ...meminta ahli keluarga untuk membeli sayur | ...meminta ahli keluarga untuk membeli sayur | ...meminta ahli keluarga untuk membeli sayur | Saya yakin saya boleh meminta ahli keluarga membeli sayur | Saya yakin saya boleh meminta ahli keluarga membeli sayur | Saya yakin saya boleh meminta ahli keluarga membeli sayur |
| VSE17 | ...eat vegetables during a hot meal | ...makan sayur semasa makan utama | ...makan sayur semasa makan utama (sarapan, makan tengah hari dan makan malam) | ...makan sayur semasa makan utama (sarapan, makan tengah hari dan makan malam) | Saya yakin saya boleh makan sayur semasa makan utama (sarapan, makan tengah hari dan makan malam) | Saya yakin saya boleh makan sayur semasa makan utama (sarapan, makan tengah hari dan makan malam) | Saya yakin saya boleh makan sayur semasa makan utama (sarapan, makan tengah hari dan makan malam) |
| VSE_ADD | Suggestion: additional questions during expert reviewing and content validity - need to be considered by the research team | | ...makan sayur sebanyak tiga atau lebih hidangan dalam sehari | ...makan sayur sebanyak tiga atau lebih hidangan dalam sehari (.*..eat in three or more servings of vegetables per day*) | Saya yakin saya boleh makan sayur sebanyak tiga atau lebih hidangan dalam sehari (.*..eat in three or more servings of vegetables per day*) | .Saya yakin saya boleh makan sayur sebanyak tiga atau lebih hidangan dalam sehari (.*..eat in three or more servings of vegetables per day*) | Retained only for exploratory item set sensitivity; not included in the primary FSE-17/VSE-17 scale  Saya yakin saya boleh makan sayur sebanyak tiga atau lebih hidangan dalam sehari (.*..eat in three or more servings of vegetables per day*) |

Table S3b. Source English, final Malay, and back-translated English versions of the 17-item fruit and vegetable self-efficacy scales

| **Item** | **Original version by Bannick & van der Bajl (1)** | **Malay version** | **Back-translated English version** |
| --- | --- | --- | --- |
|  | *Answer choices : (1) Uncertain (2) Almost certain (3) Certain (4) Very certain* | Pilihan jawapan :  (1) Tidak pasti, (2) Hampir pasti,  (3) Pasti, (4) Sangat pasti | *Answer choices : (1) Uncertain (2) Almost certain (3) Certain (4) Very certain* |
|  | **I am certain that I can** | **Saya yakin saya boleh** | **I am confident that I can** |
| FSE01 | ...eat fruit as a snack | ... makan buah sebagai makanan ringan/snek | ... . eat fruit as a snack. |
| FSE02 | ...eat fruit if I have to prepare it myself | ... makan buah jika saya perlu sediakan sendiri seperti membasuh, mengupas, memotong dan menghidang | ... . eat fruit if I have to prepare it myself, such as by washing, peeling, cutting, and serving it |
| FSE03 | ...eat fruit during lunch | ... makan buah ketika makan tengah hari | ... . eat fruit during lunch. |
| FSE04 | ...eat fruit if my parents are not there | ... makan buah jika ibu bapa tidak bersama-sama saya | ... . eat fruit if my parents are not with me. |
| FSE05 | ...eat fruit as a snack instead of a biscuit | ... makan buah sebagai makanan ringan/ snek menggantikan biskut | ... . eat fruit as a snack instead of biscuits. |
| FSE06 | ...eat fruit during a meal at home | ... makan buah ketika makan di rumah | ... . eat fruit during a meal at home. |
| FSE07 | ...eat fruit during breakfast | ... makan buah ketika sarapan | ... . eat fruit at breakfast. |
| FSE08 | ...eat fruit even if no one else is eating fruit | ... makan buah walaupun tiada orang lain makan buah | ... . eat fruit even if no one else is eating fruit. |
| FSE09 | ...eat fruit as a snack instead of a .dy bar | ... makan buah sebagai snek/makanan ringan menggantikan gula-gula/ coklat | ... . eat fruit as a snack instead of sweets or chocolate (bar). |
| FSE10 | ...eat fruit when eating together with my friends | ... makan buah ketika bersama kawan-kawan | ... . eat fruit when I am with my friends. |
| FSE11 | ...eat fruit as a snack instead of crisps | ...makan buah sebagai snek/ makanan ringan menggantikan kerepek | ... . eat fruit as a snack instead of crisps. |
| FSE12 | ...eat two or more pieces of fruit per day | ... makan buah dua atau lebih hidangan sehari (rujuk maklumat berkaitan hidangan) | ... . eat 2 or more servings of fruit per day (see the serving information). |
| FSE13 | ...eat fruit in social situations, such as at a party, if it is available | ... makan buah di majlis/ kenduri jika ada | ... . eat fruit at a social event or feast if it is available. |
| FSE14 | ...eat fruit as dessert after a hot meal | ... makan buah sebagai pencuci mulut selepas makan | ... . eat fruit as a dessert after a meal. |
| FSE15 | ...remember to take fruit to school as a snack | ...ingat bawa buah ke sekolah sebagai snek | ... remember to bring fruit to school as a snack. |
| FSE16 | ...buy fruit if there is none in the house | ... beli buah jika tiada buah di rumah | ... buy fruit if there is none at home. |
| FSE17 | ...ask someone in my family to buy fruit | ... minta ahli keluarga belikan buah | ... . ask a family member to buy fruit for me. |
|  |  |  |  |
| VSE01 | ...eat vegetables as a snack | .... makan sayur sebagai snek/ makanan ringan (*contoh timun, tomato ceri, daun bayam goreng rangup, cendawan enoki goreng rangup, lobak merah*) | .... eat vegetables as a snack (*e.g., cucumber, cherry tomatoes, crispy fried spinach leaves, crispy fried enoki mushrooms, or carrots)*. |
| VSE02 | ...eat vegetables even if no one else is eating vegetables | .... makan sayur walaupun tiada sesiapa makan sayur | .... eat vegetables even if no one else is eating vegetables. |
| VSE03 | ...eat vegetables during lunch | .... makan sayur ketika makan tengah hari | .... eat vegetables during lunch. |
| VSE04 | ...eat vegetables if I have to prepare them myself | .... makan sayur jika saya perlu sediakannya sendiri seperti membasuh memotong, memasak sayur | .... eat vegetables if I have to prepare them myself, such as by washing, cutting, or cooking them. |
| VSE05 | ...eat vegetables if my parents are not there | .... makan sayur jika ibu bapa tidak bersama-sama saya | .... eat vegetables if my parents are not with me. |
| VSE06 | ...eat vegetables when eating together with my friends | .... makan sayur ketika makan bersama kawan-kawan | .... eat vegetables when eating with my friends. |
| VSE07 | ...eat vegetables as a snack instead of a biscuit | .... makan sayur sebagai snek bukan biskut | .... eat vegetables as a snack instead of biscuits. |
| VSE08 | ..eat vegetables in social situations, such as at a party, if available | .... makan sayur dimajlis keraian / kenduri jika disediakan | .... eat vegetables at a social event or feast if they are provided. |
| VSE09 | ...eat vegetables as a snack instead of crisps | .... makan sayur sebagai snek/ makanan ringan menggantikan kerepek | .... eat vegetables as a snack instead of crisps. |
| VSE10 | ...eat vegetables as a snack instead of a candy bar | .... makan sayur sebagai snek/makanan ringan menggantikan coklat | .... eat vegetables as a snack instead of chocolate. |
| VSE11 | ...remember to take vegetables to school as a snack | .... ingat untuk bawa sayur ke sekolah sebagai snek/ makanan ringan | .... remember to bring vegetables to school as a snack. |
| VSE12 | ...buy vegetables if there are none in the house | ... boleh beli sayur jika tiada sayur di rumah | .... buy vegetables if there are no vegetables at home. |
| VSE13 | ...ask someone in my family to prepare vegetables for a hot meal | .... minta ahli keluarga masak sayur untuk hidangan utama | ... ask a family member to cook some vegetables for me to eat. |
| VSE14 | ...eat vegetables during a meal at home | .... makan sayur ketika makan di rumah | .... eat vegetables during a meal at home. |
| VSE15 | ...eat four or more serving spoons of vegetables per serving | .... makan sayur empat atau lebih sudu setiap hidangan (kali makan) | .... eat four or more spoonfuls of vegetables at each meal. |
| VSE16 | ...ask someone in my family to buy vegetables | .... meminta ahli keluarga membeli sayur | .... ask a family member to buy vegetables. |
| VSE17 | ...eat vegetables during a hot meal | .... makan sayur semasa makan utama (sarapan, makan tengah hari dan makan malam) | .... eat vegetables during main meals (breakfast, lunch, and dinner). |
