# Supplementary material for: Cross-cultural adaptation and initial psychometric evaluation of the fruit and vegetable self-efficacy scales among secondary school adolescents in Selangor, Malaysia
Source: Front Public Health. 2026 Sep 29;14:1920784. doi: 10.3389/fpubh.2026.1920784 (PMC13658580; doi:10.3389/fpubh.2026.1920784)
Supplement: Supplementary file 5 [file Table_4.docx]

**Supplementary 4**

Table S4a. Characteristics of the expert panel participating in content-validity assessment

| Expert role | Content validation (n = 9) | Years of professional experience (range) |
| --- | --- | --- |
| Health education officers | 3 | 10-20 |
| Nutrition officer | 1 | 10 |
| Instrument development specialists | 2 | 10-20 |
| Educator | 1 | 5 |
| Academic researchers (public health/health behavior) | 2 | 15-20 |

Table S4b. Rating scales and dichotomous coding used for the Content Validity Index

and Content Validity Ratio

|  | **1** | **2** | **3** | **4** |
| --- | --- | --- | --- | --- |
| CVI | not relevant at all | almost relevant | relevant | very relevant |
| CVR | not essential at all | almost essential | essential | very essential |
| **Rating** | **0** | | **1** | |

Note. CVI = Content Validity Index; CVR = Content Validity Ratio. Ratings of 1 and 2 were coded as 0, whereas ratings of 3 and 4 were coded as 1 for the respective CVI and CVR calculations.

Table S4c.Content Validity Index and Content Validity Ratio for the Fruit Self-Efficacy scale

|  | **Relevant** | | **Essential** | |
| --- | --- | --- | --- | --- |
|  | **I-CVI** | **UA** | **I-CVR** | **UA** |
| FSE01 | 1.00 | 1.00 | 1.00 | 1.00 |
| FSE02 | 1.00 | 1.00 | 1.00 | 1.00 |
| FSE03 | 1.00 | 1.00 | 1.00 | 1.00 |
| FSE04 | 1.00 | 1.00 | 1.00 | 1.00 |
| FSE05 | 1.00 | 1.00 | 1.00 | 1.00 |
| FSE06 | 1.00 | 1.00 | 1.00 | 1.00 |
| FSE07 | 1.00 | 1.00 | 1.00 | 1.00 |
| FSE08 | 1.00 | 1.00 | 1.00 | 1.00 |
| FSE09 | 1.00 | 1.00 | 1.00 | 1.00 |
| FSE10 | 1.00 | 1.00 | 1.00 | 1.00 |
| FSE11 | 1.00 | 1.00 | 1.00 | 1.00 |
| FSE12 | 1.00 | 1.00 | 1.00 | 1.00 |
| FSE13 | 1.00 | 1.00 | 1.00 | 1.00 |
| FSE14 | 1.00 | 1.00 | 1.00 | 1.00 |
| FSE15 | 1.00 | 1.00 | 1.00 | 1.00 |
| FSE16 | 0.89 | 0.00 | 0.78 | 0.00 |
| FSE17 | 1.00 | 1.00 | 1.00 | 1.00 |
|  |  |  |  |  |
|  | **16.89/17** | **16/17** | **16.78/17** | **16/17** |
|  | **S-CVI/Ave**  **= 0.99** | **S-CVI/UA**  **=0.94** | **S-CVR/Ave = 0.99** | **S-CVR/UA**  **=0.94** |

**Abbreviations:** I-CVI, item-level Content Validity Index; I-CVR, item-level Content Validity Ratio; S-CVI/Ave, scale-level Content Validity Index calculated using the average method; S-CVI/UA, proportion of source items receiving unanimous relevance ratings; S-CVR/Ave, mean item-level Content Validity Ratio across the 17 source items; S-CVR/UA, proportion of source items receiving unanimous essentiality ratings. I-CVI was calculated as the proportion of experts who assigned a relevance rating of 3 or 4, divided by the total number of experts. I-CVR was calculated as $\left( N_{e}-N/2 \right)/\left( N/2 \right)$, where $N_{e}$is the number of experts rating an item as essential, and $N$is the total number of experts. For a panel of nine experts, I-CVI and I-CVR values ≥0.78 were considered acceptable. An S-CVI/Ave ≥0.90 was considered acceptable. S-CVR/Ave was interpreted descriptively, with values closer to 1.00 indicating stronger scale-level agreement regarding item essentiality. In the UA columns, 1 indicates unanimous agreement among all nine experts, and 0 indicates that unanimity was not achieved (1–5)

Table S4d. Content Validity Index and Content Validity Ratio for the Vegetable Self-Efficacy scale

|  | **Relevant** | | **Essential** | |
| --- | --- | --- | --- | --- |
|  | **I-CVI** | **UA** | **I-CVR** | **UA** |
| VSE01 | 1.00 | 1.00 | 1.00 | 1.00 |
| VSE02 | 1.00 | 1.00 | 1.00 | 1.00 |
| VSE03 | 1.00 | 1.00 | 1.00 | 1.00 |
| VSE04 | 1.00 | 1.00 | 1.00 | 1.00 |
| VSE05 | 1.00 | 1.00 | 1.00 | 1.00 |
| VSE06 | 1.00 | 1.00 | 1.00 | 1.00 |
| VSE07 | 0.78 | 0.00 | 0.78 | 0.00 |
| VSE08 | 1.00 | 1.00 | 1.00 | 1.00 |
| VSE09 | 0.89 | 0.00 | 1.00 | 1.00 |
| VSE10 | 0.78 | 0.00 | 0.78 | 0.00 |
| VSE11 | 0.78 | 0.00 | 0.78 | 0.00 |
| VSE12 | 1.00 | 1.00 | 1.00 | 1.00 |
| VSE13 | 1.00 | 1.00 | 1.00 | 1.00 |
| VSE14 | 1.00 | 1.00 | 1.00 | 1.00 |
| VSE15 | 1.00 | 1.00 | 1.00 | 1.00 |
| VSE16 | 1.00 | 1.00 | 1.00 | 1.00 |
| VSE17 | 0.89 | 0.00 | 1.00 | 1.00 |
|  |  |  |  |  |
|  | 16.11/17 | 12/17 | 16.33/17 | 14/17 |
|  | **S-CVI/Ave**  **=0.95** | **S-CVI/UA**  **=0.71** | **S-CVR/Ave = 0.96** | **S-CVR/UA**  **=0.82** |

**Abbreviations:** I-CVI, item-level Content Validity Index; I-CVR, item-level Content Validity Ratio; S-CVI/Ave, scale-level Content Validity Index calculated using the average method; S-CVI/UA, proportion of source items receiving unanimous relevance ratings; S-CVR/Ave, mean item-level Content Validity Ratio across the 17 source items; S-CVR/UA, proportion of source items receiving unanimous essentiality ratings. I-CVI was calculated as the proportion of experts who assigned a relevance rating of 3 or 4, divided by the total number of experts. I-CVR was calculated as $\left( N_{e}-N/2 \right)/\left( N/2 \right)$, where $N_{e}$is the number of experts rating an item as essential and $N$is the total number of experts. For a panel of nine experts, I-CVI and I-CVR values ≥0.78 were considered acceptable. An S-CVI/Ave ≥0.90 was considered acceptable. S-CVR/Ave was interpreted descriptively, with values closer to 1.00 indicating stronger scale-level agreement regarding item essentiality. In the UA columns, 1 indicates unanimous agreement among all nine experts, and 0 indicates that unanimity was not achieved. (1–5)

Table S4e. Summary of expert feedback and actions taken

| Expert Comment | Action taken |
| --- | --- |
| Several FSE items concerning fruit consumption as a snack (FSE01, FSE05, FSE09, and FSE11) were perceived as conceptually overlapping because they could be interpreted as representing similar behaviours. Comparable contextual overlap was also noted among some VSE snack-related items. | All source items were retained to preserve the conceptual breadth and comparability of the original instruments. Potential item redundancy was subsequently examined through item-level analyses and will require further evaluation in independent psychometric studies. |
| In the Malaysian cultural context, particularly among Malay adolescents, consuming fruit or vegetables at breakfast was perceived as relatively uncommon (FSE07 and VSE17). | The item wording was retained to maintain conceptual equivalence with the source instruments while acknowledging its potentially limited contextual relevance. Item performance was subsequently examined in the main psychometric evaluation. |
| Some VSE items were perceived as less contextually relevant and were considered to require further psychometric evaluation. | Their performance was examined through item-level response distributions, factor loadings, CFA global-fit indices, and reliability analyses. Reliability and item-level evidence were interpreted separately from global model fit, and no item was removed solely on the basis of one statistical indicator. |
| Experts proposed two context-specific additional items: “buy fruit while undertaking leisure activities” for FSE and “eat three or more servings of vegetables per day” for VSE. | The proposed items were evaluated through exploratory item-set sensitivity analyses. Neither item demonstrated sufficient incremental psychometric benefit to be incorporated into the primary 17-item scales. |

References

1.	Yusoff MSB. ABC of Content Validation and Content Validity Index Calculation. Education in Medicine Journal. 2019;11(2):49–54. doi:10.21315/eimj2019.11.2.6.

2.	Zamanzadeh V, Ghahramanian A, Rassouli M, Abbaszadeh A, Alavi-Majd H, Nikanfar AR. Design and Implementation Content Validity Study: Development of an instrument for measuring Patient-Centered Communication. J Caring Sci. 2015;4(2):165–78. doi:10.15171/jcs.2015.017.

3.	Lynn MR. Determination and quantification of content validity. Nursing Research [Internet]. 1986. p. 382–6. Available from: http://ijoh.tums.ac.ir/index.php/ijoh/article/view/26.

4.	Lawshe CH. A quantitative approach to content validity. Pers Psychol. 1975;28(4):563–575.doi:10.1111/j.1744-6570.1975.tb01393.x.

5.	Ayre C, Scally AJ. Critical values for Lawshe’s content validity ratio: Revisiting the original methods of calculation. Measurement and Evaluation in Counseling and Development. 2014;47(1):79–86. doi:10.1177/0748175613513808.
