# Supplementary material for: Cross-cultural adaptation and initial psychometric evaluation of the fruit and vegetable self-efficacy scales among secondary school adolescents in Selangor, Malaysia
Source: Front Public Health. 2026 Sep 29;14:1920784. doi: 10.3389/fpubh.2026.1920784 (PMC13658580; doi:10.3389/fpubh.2026.1920784)
Supplement: Supplementary file 6 [file Table_5.docx]

**Supplementary 5**

Following the cognitive debriefing process, quantitative face validity was evaluated among 10 adolescents using the Face Validity Index (FVI) approach.

Table S5a. Fruit self-efficacy (FSE) face validity index (FVI)

|  | Clarity | | Comprehensibility | |
| --- | --- | --- | --- | --- |
|  | I-FVI | UA | I-FVI | UA |
| FSE01 | 0.90 | 0.00 | 0.90 | 0.00 |
| FSE02 | 0.70 | 0.00 | 0.90 | 0.00 |
| FSE03 | 1.00 | 1.00 | 1.00 | 1.00 |
| FSE04 | 1.00 | 1.00 | 1.00 | 1.00 |
| FSE05 | 1.00 | 1.00 | 1.00 | 1.00 |
| FSE06 | 0.80 | 0.00 | 0.90 | 0.00 |
| FSE07 | 1.00 | 1.00 | 1.00 | 1.00 |
| FSE08 | 1.00 | 1.00 | 0.90 | 0.00 |
| FSE09 | 1.00 | 1.00 | 1.00 | 1.00 |
| FSE10 | 1.00 | 1.00 | 1.00 | 1.00 |
| FSE11 | 1.00 | 1.00 | 1.00 | 1.00 |
| FSE12 | 1.00 | 1.00 | 1.00 | 1.00 |
| FSE13 | 1.00 | 1.00 | 1.00 | 1.00 |
| FSE14 | 1.00 | 1.00 | 1.00 | 1.00 |
| FSE15 | 1.00 | 1.00 | 1.00 | 1.00 |
| FSE16 | 1.00 | 1.00 | 0.90 | 0.00 |
| FSE17 | 1.00 | 1.00 | 1.00 | 1.00 |
| FSE-ADD (exploratory) | 1.00 | 1.00 | 1.00 | 1.00 |
|  |  |  |  |  |
|  | 17.40/18 | 15.00/18 | 17.50/18 | 13.00/18 |
|  | **S-FVI/Ave**  **= 0.97** | **S-FVI/UA**  **=0.83** | **S-FVI/Ave**  **= 0.97** | **S-FVI/UA**  **=0.72** |

**Abbreviations:** I-FVI, Item-level Face Validity Index; S-FVI/Ave, Scale-level Face Validity Index calculated using the average method; S-FVI/UA, Scale-level Face Validity Index based on universal agreement. An I-FVI value of ≥0.80 was considered acceptable.(1–5)

Table S5b. Vegetables self-efficacy (VSE) face validity index (FVI)

|  | Clarity | | Comprehensibility | |
| --- | --- | --- | --- | --- |
|  | I-FVI | UA | I-FVI | UA |
| VSE01 | 1.00 | 1.00 | 0.90 | 0.00 |
| VSE02 | 0.90 | 0.00 | 0.90 | 0.00 |
| VSE03 | 1.00 | 1.00 | 1.00 | 1.00 |
| VSE04 | 0.80 | 0.00 | 1.00 | 1.00 |
| VSE05 | 1.00 | 1.00 | 1.00 | 1.00 |
| VSE06 | 1.00 | 1.00 | 1.00 | 1.00 |
| VSE07 | 0.90 | 0.00 | 0.90 | 0.00 |
| VSE08 | 1.00 | 1.00 | 1.00 | 1.00 |
| VSE09 | 1.00 | 1.00 | 1.00 | 1.00 |
| VSE10 | 0.90 | 0.00 | 0.90 | 0.00 |
| VSE11 | 1.00 | 1.00 | 0.90 | 0.00 |
| VSE12 | 1.00 | 1.00 | 1.00 | 1.00 |
| VSE13 | 0.80 | 0.00 | 1.00 | 1.00 |
| VSE14 | 1.00 | 1.00 | 1.00 | 1.00 |
| VSE15 | 0.90 | 0.00 | 0.90 | 0.00 |
| VSE16 | 1.00 | 1.00 | 1.00 | 1.00 |
| VSE17 | 0.90 | 0.00 | 1.00 | 1.00 |
| VSE-ADD (exploratory) | 1.00 | 1.00 | 0.90 | 0.00 |
|  |  |  |  |  |
|  | 17.10/18 | 11.00/18 | 17.30/18 | 11.00/18 |
|  | **S-FVI/Ave**  **= 0.95** | **S-VI/UA**  **=0.61** | **S-FVI/Ave**  **= 0.96** | **S-FVI/UA**  **=0.61** |

**Abbreviations:** I-FVI, Item-level Face Validity Index; S-FVI/Ave, Scale-level Face Validity Index calculated using the average method; S-FVI/UA, Scale-level Face Validity Index based on universal agreement. An I-FVI value of ≥0.80 was considered acceptable. (1–5)

Table S5c. Reassessment of the clarity and comprehensibility of the FVSE among low rater participants

A. Clarity

|  | A1 | | | | A6 | | | | A10 | | | |
| --- | --- | --- | --- | --- | --- | --- | --- | --- | --- | --- | --- | --- |
|  | Assessment | | Reassessment | | Assessment | | Reassessment | | Assessment | | Reassessment | |
| FSE01 |  |  | 4 | 1 | 2 | 0 | 4 | 1 | 2 | 0 | 3 | 1 |
| FSE02 | 2 | 0 | 3 | 1 | 2 | 0 | 4 | 1 | 2 | 0 | 3 | 1 |
| FSE06 | 2 | 0 | 3 | 1 |  |  |  |  | 1 | 0 | 4 | 1 |
|  |  |  |  |  |  |  |  |  |  |  |  |  |
| VSE02 | 2 | 0 | 3 | 1 |  |  |  |  |  |  |  |  |
| VSE04 | 2 | 0 | 4 | 1 | 2 | 0 | 3 | 1 | 2 | 0 | 3 | 1 |
| VSE07 | 2 | 0 | 3 | 1 |  |  |  |  |  |  |  |  |
| VSE10 | 2 | 0 | 3 | 1 |  |  |  |  |  |  |  |  |
| VSE13 | 2 | 0 | 3 | 1 |  |  |  |  |  |  |  |  |
| VSE15 | 2 | 0 | 4 | 1 |  |  |  |  |  |  |  |  |
| VSE16 | 2 | 0 | 4 | 1 |  |  |  |  |  |  |  |  |

B. Comprehensiveness

| Q/ RATER | | A1 | | | | | | | A6 | | | | |
| --- | --- | --- | --- | --- | --- | --- | --- | --- | --- | --- | --- | --- | --- |
|  | | Assessment | | | | Reassessment | | | Assessment | | | Reassessment | |
| FSE01 | | 4 | | 1 | |  |  | | 2 | | 0 | 4 | 1 |
| FSE02 | | 2 | | 0 | | 4 | 1 | |  | |  |  |  |
| FSE06 | | 2 | | 0 | | 4 | 1 | |  | |  |  |  |
| FSE08 | | 2 | | 0 | | 4 | 1 | |  | |  |  |  |
| FSE16 | | 2 | | 0 | | 4 | 1 | |  | |  |  |  |
|  |  | | | |  | | | | |  |  |  |  |
| VSE01 | 2 | | 0 | | 4 | | | 1 | |  |  |  |  |
| VSE02 | 2 | | 0 | | 4 | | | 1 | |  |  |  |  |
| VSE07 | 2 | | 0 | | 4 | | | 1 | |  |  |  |  |
| VSE10 | 2 | | 0 | | 4 | | | 1 | |  |  |  |  |
| VSE11 | 2 | | 0 | | 4 | | | 1 | |  |  |  |  |
| VSE15 | 2 | | 0 | | 4 | | | 1 | |  |  |  |  |

Table S5d. Difficulties of participant A1 (age 13, Boy)

| Difficulties | Action |
| --- | --- |
| Words   - Hidangan (servings) - Waktu makan utama (main meals) - Pencuci mulut (desserts) | Cognitive debriefing was conducted to identify any difficulties Participant A1 experienced and to reassess items that received low ratings. Participant A1 was asked to complete a paper-based questionnaire, explain any items that were difficult to understand, and provide feedback on questions requiring further improvement. (refer also to supplementary 3) |
| Did not understand the need for the question well. |  |

**Face validity:**

Qualitative face-validity assessment

Instrument	: Fruit and Vegetable Intake Self-Efficacy Questionnaire, 18-item, Malay version	(FVSE_M0.3)

Participant	: Adolescents

Number of participants	: 5 (3 Girls, 2 Boys)

Age	: 12-16

Date	: 29 December 2024

Objective	: To evaluate the clarity, comprehensibility, terminology, and sentence structure of the Malay translation.

Procedure	: Each item was read aloud and reviewed individually. Participants were asked whether the item was clear and understandable and were invited to explain its meaning in their own words. When an item was perceived as unclear or difficult to understand, participants were asked to identify the specific words, phrases, or contextual aspects that caused the difficulty.

| **Item** | **Original Item (English)** | | **Malay translation (FVSE_M0.3)** | | **Comprehensive** | **Clarity** | **Key Issues Identified by Participants** | **Revision Rationale / Action Taken** |
| --- | --- | --- | --- | --- | --- | --- | --- | --- |
|  | *Answer choices :  (1) Uncertain (2) Almost ceartain (3) Certain (4) Very certain* | | Pilihan jawapan :  (1) Tidak pasti (2) Hampir pasti (3) Pasti (4) Sangat pasti | |  |  | **Pilihan jawapan sepatutnya contoh – tidak pasti yakin, hampir pasti yakin, yakin, sangat yakin** | **Kekalkan plihan jawapan yang telah diterjemahkan** |
| *S0* | I am certain that I can… | | Saya yakin saya boleh... | |  |  |  |  |
|  | |  | | **Fruit Self-efficacy / Efikasi kendiri pengambilan buah** | | | | |
| FSE01 | ...eat fruit as a snack | | …makan buah sebagai snek/ makanan ringan | | Good, understand well | Good |  |  |
| FSE02 | ...eat fruit if I have to prepare it myself | | ...makan buah jika saya perlu menyediakannya sendiri | | Can understand | Good but - what type of preparation to do | menyediakannya  Give example “what kind of menyediakan” | Used simple word “sediakan”  Give example – washing, kupas and cutting – put in plate |
| FSE03 | ...eat fruit during lunch | | ...makan buah semasa makan tengah hari | | Good, understand well | Good |  |  |
| FSE04 | ...eat fruit if my parents are not with me | | ...makan buah jika ibu bapa tidak ada bersama saya | | Good, understand well | Good- father and mother need to together? | Refine the sentences | Delete ‘ada” |
| FSE05 | ...eat fruit as a snack instead of a biscuit | | ...makan buah sebagai snek/ makanan ringan menggantikan biskut | | Good, understand well | Good – anything type of biscuits? (1) |  | Explanation – for clarification |
| FSE06 | ...eat fruit during a meal at home | | ...makan buah ketika makan di rumah | | Good, understand well | Good |  |  |
| FSE07 | ...eat fruit during breakfast | | ...makan buah ketika sarapan | | Good, understand well | Good |  |  |
| FSE08 | ...eat fruit even if no one else is eating fruit | | ...makan buah walaupun tiada orang lain makan buah | | Good, understand well | Good |  |  |
| FSE09 | ...eat fruit as a snack instead of a candy bar | | ...makan buah sebagai snek/ makanan ringan menggantikan coklat | | Good, understand well | Good |  |  |
| FSE10 | ...eat fruit with my friends | | ...makan buah dengan kawan-kawan | | Good, understand well | Good |  |  |
| FSE11 | ...eat fruit as a snack instead of crisps | | ...makan buah sebagai snek / makanan ringan menggantikan kerepek | | Good, understand well | Good – “Kerepek”- includes Twister, Jack n Jill, Mr. Potato? - |  | Explanation – for clarification |
| FSE12 | ...eat two or more pieces of fruit per day | | ...makan buah dua atau lebih hidangan sehari | | Good, understand well – “serving” - | Good |  | Explanation – for clarification |
| FSE13 | ...eat fruit in social situations, such as at a party, if it is available | | ...makan buah dimajlis/ kenduri/ pesta, jika ada | | Can understand – What does " pesta " mean? – in pesta – there is no food served in pesta | Need amendment | “ pesta” = refers to an event or celebration held on a large scale. | Delete “Pesta” |
| FSE14 | ...eat fruit as a dessert after a hot meal | | ...makan buah sebagai pencuci mulut selepas makan | | Can understand – What does " hot meal " mean in Malay: " makan panas? Makan utama?” | Need amendment | What is “hot meal?” | Do not direct translation of hot meal |
| FSE15 | ...remember to bring fruit to school as a snack | | ... ingat untuk bawa buah ke sekolah sebagai snek | | Good, understand well | Good |  |  |
| FSE16 | ...buy fruit if there is none in the house | | ...beli buah jika tiada di rumah | | Good, understand well | Good – Refine sentences to be clearer | .....tiada buah di rumah | Refine sentence |
| FSE17 | ...ask someone in my family to buy fruit | | ...minta ahli keluarga membelikan buah | | Good, understand well | Good |  |  |
| FSE-ADD | .*..buy fruit when I am out or doing activities* | | beli buah semasa berjalan-jalan /beriadah | | Good, understand well | Good |  |  |
|  | |  | | ***Vegetable Self-efficacy / Efikasi kendiri pengambilan sayur** | | | | |
| VSE01 | ...eat vegetables as a snack | | ...makan sayur sebagai snek/ makanan ringan | | Good, understand well | Good- What kind of vegetable can be snack? | What kind of vegetables can be snack? | Explanation – for clarification |
| VSE02 | ...eat vegetables even if no one else is eating vegetables | | ...makan sayur walaupun tiada sesiapa makan sayur | | Good, understand well | Good |  |  |
| VSE03 | ...eat vegetables during lunch | | ...makan sayur semasa makan tengah hari | | Good, understand well | Good |  |  |
| VSE04 | ...eat vegetables if I have to prepare it myself | | ...makan sayur jika saya perlu sediakannya sendiri | | Same as FSE comment | Explain – what kind of preparation | What is the meaning of preparation? | Give an example of preparation – washing, cutting, cooking and serving |
| VSE05 | ...eat vegetables if my parents are not with me | | ...makan sayur walaupun ibu bapa tidak ada bersama dengan saya. | | Good, understand well | Good | ....tidak MAKAN bersama dengan saya..... | Acceptable |
| VSE06 | ...eat vegetables when eating together with my friends | | ...makan sayur apabila makan bersama kawan-kawan | | Good, understand well | Good |  |  |
| VSE07 | ...eat vegetables as a snack instead of a biscuit | | ...makan sayur sebagai snek bukan biskut | | Good, understand well | Good |  |  |
| VSE08 | ...eat vegetables in events such as at a party, if available | | ...makan sayur dimajlis/ kenduri jika disediakan | | Good, understand well | Good |  |  |
| VSE09 | ...eat vegetables as a snack instead of crisps | | ...makan sayur sebagai snek/ makanan ringan menggantikan kerepek | | Good, understand well | Good – “Kerepek”- includes Twister, Jack n Jill, Mr. Potato? - |  | Explanation – for clarification |
| VSE10 | ...eat vegetables as a snack instead of a candy bar | | ...makan sayur sebagai snek/makanan ringan menggantikan coklat | | Good, understand well | Confusing-How can vegetables be a snack instead of a chocolate bar |  | Explanation – for clarification |
| VSE11 | ...remember to take vegetables to school as a snack | | ...ingat untuk bawa sayur ke sekolah sebagai snek/ makanan ringan | | Good, understand well | Good- same as no 1 question |  |  |
| VSE12 | ...buy vegetables if there are none in the house | | ...beli sayur jika tiada sayur di rumah | | parent buys vegetables (not their duty) | |  |  |
| VSE13 | ...ask someone in my family to prepare vegetables for a hot meal | | ...minta ahli keluarga saya masak sayur untuk makan panas-panas | | Can understand – What does " hot meal " mean in Malay: " makan panas? Makan utama?” | Need amendment | “makan panas-panas?” | Delete “panas-panas” |
| VSE14 | ...eat vegetables during a meal at home | | ...makan sayur semasa makan di rumah | | Good, understand well | Good |  |  |
| VSE15 | ...eat four or more serving spoons of vegetables per serving | | ...makan sayur empat atau lebih sudu hidangan setiap kali makan | | Need amendment -explain hidangan | Need amendment - too many " four or more spoons " (need to follow MDG) | Delete “hidangan” | Delete “hidangan in malay version” |
| VSE16 | ...ask someone in my family to buy vegetables | | ...meminta ahli keluarga untuk membeli sayur | | Good, understand well | Good |  |  |
| VSE17 | ...eat vegetables during main meals (breakfast, lunch and dinner) | | ...makan sayur semasa makan utama (sarapan, makan tengahari dan makan malam) | | Good, understand well | Good |  |  |
| VSE-ADD | .*..eat in three or more servings of vegetables per day* | | ..makan sayur sebanyak tiga atau lebih hidangan dalam sehari | | Good, understand well | Good |  |  |

**References**

1.	Yusoff MSB. ABC of Content Validation and Content Validity Index Calculation. Education in Medicine Journal. 2019;11(2):49–54. doi:10.21315/eimj2019.11.2.6

2.	Zamanzadeh V, Ghahramanian A, Rassouli M, Abbaszadeh A, Alavi-Majd H, Nikanfar AR. Design and Implementation Content Validity Study: Development of an instrument for measuring Patient-Centered Communication. J Caring Sci. 2015;4(2):165–78. doi:10.15171/jcs.2015.017 PubMed PMID: 26161370.

3.	Lynn MR. Determination and quantification of content validity. Nursing Research [Internet]. 1986. p. 382–6. Available from: http://ijoh.tums.ac.ir/index.php/ijoh/article/view/26 PubMed PMID: 28668634.

4.	Lawshe CH. A quantitative approach to content validity”.Personnel Psychology. Pers Psychol. 1975;28:563–75.

5.	Ayre C, Scally AJ. Critical values for Lawshe’s content validity ratio: Revisiting the original methods of calculation. Measurement and Evaluation in Counseling and Development. 2014;47(1):79–86. doi:10.1177/0748175613513808
