# Supplementary material for: Cross-cultural adaptation and initial psychometric evaluation of the fruit and vegetable self-efficacy scales among secondary school adolescents in Selangor, Malaysia
Source: Front Public Health. 2026 Sep 29;14:1920784. doi: 10.3389/fpubh.2026.1920784 (PMC13658580; doi:10.3389/fpubh.2026.1920784)
Supplement: Supplementary file 7 [file Table_7.docx]

## Supplement Table S7

## Table S7a. Internal-consistency reliability of the original 17-item Fruit Self-Efficacy scale

| **Item/scale** | **McDonald’s ω Estimate (95% CI)** | **Cronbach’s α Estimate (95% CI)** | **Corrected item–total correlation (item–rest correlation) (95% CI)** |
| --- | --- | --- | --- |
| **Overall scale** | **.943 (.919–.959)** | **.943 (.918–.959)** | **—** |
| FSE01 | .938 (.912–.956) | .938 (.911–.955) | .749 (.637–.829) |
| FSE02 | .941 (.915–.958) | .940 (.914–.957) | .643 (.501–.757) |
| FSE03 | .939 (.913–.956) | .939 (.912–.956) | .726 (.616–.814) |
| FSE04 | .939 (.914–.956) | .939 (.913–.956) | .694 (.575–.786) |
| FSE05 | .938 (.912–.956) | .938 (.912–.955) | .756 (.648–.834) |
| FSE06 | .941 (.916–.958) | .941 (.915–.957) | .611 (.479–.723) |
| FSE07 | .940 (.914–.957) | .939 (.913–.956) | .697 (.602–.778) |
| FSE08 | .940 (.914–.957) | .939 (.913–.956) | .693 (.579–.790) |
| FSE09 | .941 (.915–.958) | .940 (.914–.957) | .640 (.519–.739) |
| FSE10 | .940 (.914–.957) | .939 (.914–.956) | .682 (.549–.782) |
| FSE11 | .940 (.914–.957) | .939 (.913–.957) | .679 (.578–.760) |
| FSE12 | .939 (.913–.956) | .938 (.912–.955) | .728 (.610–.818) |
| FSE13 | .940 (.914–.957) | .940 (.913–.956) | .675 (.545–.776) |
| FSE14 | .940 (.914–.957) | .939 (.914–.956) | .697 (.570–.793) |
| FSE15 | .940 (.913–.957) | .939 (.912–.957) | .700 (.612–.774) |
| FSE16 | .942 (.916–.959) | .942 (.915–.959) | .548 (.436–.648) |
| FSE17 | .941 (.915–.958) | .940 (.914–.957) | .640 (.486–.759) |

## Table S7b. Internal-consistency reliability of the original 17-item Vegetable Self-Efficacy scale

| **Item/scale** | **McDonald’s ω Estimate (95% CI)** | **Cronbach’s α Estimate (95% CI)** | **Corrected item–total correlation (item–rest correlation) (95% CI)** |
| --- | --- | --- | --- |
| **Overall scale** | **.955 (.942–.966)** | **.955 (.941–.966)** | **—** |
| VSE01 | .952 (.937–.964) | .951 (.936–.963) | .776 (.711–.831) |
| VSE02 | .952 (.937–.963) | .951 (.936–.963) | .772 (.681–.848) |
| VSE03 | .953 (.938–.964) | .952 (.937–.963) | .729 (.639–.802) |
| VSE04 | .953 (.939–.964) | .953 (.938–.964) | .699 (.605–.780) |
| VSE05 | .952 (.937–.963) | .951 (.936–.963) | .771 (.694–.839) |
| VSE06 | .951 (.936–.963) | .950 (.935–.962) | .830 (.774–.876) |
| VSE07 | .953 (.938–.964) | .952 (.937–.964) | .741 (.665–.807) |
| VSE08 | .952 (.937–.963) | .951 (.936–.963) | .793 (.722–.852) |
| VSE09 | .954 (.939–.965) | .953 (.938–.964) | .691 (.580–.781) |
| VSE10 | .954 (.940–.965) | .953 (.938–.964) | .681 (.588–.762) |
| VSE11 | .955 (.941–.965) | .954 (.940–.965) | .612 (.496–.716) |
| VSE12 | .955 (.941–.966) | .955 (.940–.965) | .597 (.478–.696) |
| VSE13 | .953 (.938–.964) | .952 (.937–.964) | .730 (.629–.813) |
| VSE14 | .952 (.938–.964) | .952 (.937–.963) | .753 (.667–.822) |
| VSE15 | .951 (.937–.963) | .951 (.936–.963) | .791 (.715–.852) |
| VSE16 | .954 (.940–.965) | .953 (.939–.964) | .666 (.553–.761) |
| VSE17 | .953 (.938–.964) | .952 (.937–.964) | .718 (.623–.796) |

Note. The “Overall scale” row reports scale-level reliability estimates. For each individual-item row, McDonald’s ω and Cronbach’s α represent the reliability coefficients recalculated after removal of the corresponding item. The item–rest correlation represents the correlation between the item and the total score calculated from all remaining items. McDonald’s ω was estimated using principal factor analysis (PFA), and 95% confidence intervals were obtained using non-parametric bootstrap procedures. Standardized coefficients are reported. These coefficients characterise the internal consistency of the observed scores and should not be interpreted as evidence of unidimensionality or adequate structural validity. This distinction is particularly important for the VSE-17 scale, for which the prespecified one-factor model demonstrated inadequate global fit.
