# Supplementary material for: Cross-cultural adaptation and initial psychometric evaluation of the fruit and vegetable self-efficacy scales among secondary school adolescents in Selangor, Malaysia
Source: Front Public Health. 2026 Sep 29;14:1920784. doi: 10.3389/fpubh.2026.1920784 (PMC13658580; doi:10.3389/fpubh.2026.1920784)
Supplement: Supplementary file 8 [file Table_8.docx]

Supplement Table S8

Table S8a. Item-level confirmatory factor analysis results for the Fruit Self-Efficacy (FSE) scale.

| **Indicator** | **Std. estimate** | **Std. Error** | **z-value** | **p** | **95% Confidence Interval** | | **R²** |
| --- | --- | --- | --- | --- | --- | --- | --- |
|  |  |  |  |  | **Lower** | **Upper** |  |
| FSE01 | .832 | .039 | 21.45 | < .001 | .756 | .908 | .692 |
| FSE02 | .734 | .052 | 14.11 | < .001 | .632 | .836 | .538 |
| FSE03 | .812 | .037 | 22.19 | < .001 | .740 | .884 | .659 |
| FSE04 | .824 | .042 | 19.62 | < .001 | .741 | .906 | .679 |
| FSE05 | .862 | .030 | 28.98 | < .001 | .803 | .920 | .742 |
| FSE06 | .710 | .050 | 14.13 | < .001 | .611 | .808 | .504 |
| FSE07 | .805 | .036 | 22.13 | < .001 | .733 | .876 | .647 |
| FSE08 | .841 | .037 | 22.45 | < .001 | .768 | .914 | .707 |
| FSE09 | .772 | .041 | 18.80 | < .001 | .691 | .852 | .595 |
| FSE10 | .810 | .039 | 20.65 | < .001 | .733 | .886 | .655 |
| FSE11 | .784 | .038 | 20.49 | < .001 | .709 | .859 | .615 |
| FSE12 | .838 | .038 | 21.76 | < .001 | .762 | .913 | .702 |
| FSE13 | .788 | .041 | 19.32 | < .001 | .708 | .868 | .621 |
| FSE14 | .781 | .043 | 17.97 | < .001 | .696 | .866 | .610 |
| FSE15 | .789 | .039 | 20.41 | < .001 | .713 | .864 | .622 |
| FSE16 | .647 | .060 | 10.84 | < .001 | .530 | .764 | .419 |
| FSE17 | .748 | .053 | 14.09 | < .001 | .644 | .852 | .559 |

Table S8b. Item-level confirmatory factor analysis results for the Vegetable Self-Efficacy (VSE) scale.

| **Indicator** | **Std. estimate** | **Std. Error** | **z-value** | **p** | **95% Confidence Interval** | | **R²** |
| --- | --- | --- | --- | --- | --- | --- | --- |
|  |  |  |  |  | **Lower** | **Upper** |  |
| VSE01 | .858 | .025 | 34.71 | < .001 | .810 | .907 | .736 |
| VSE02 | .837 | .032 | 26.16 | < .001 | .774 | .899 | .700 |
| VSE03 | .869 | .027 | 32.62 | < .001 | .816 | .921 | .754 |
| VSE04 | .792 | .035 | 22.34 | < .001 | .722 | .861 | .627 |
| VSE05 | .840 | .029 | 28.51 | < .001 | .782 | .897 | .705 |
| VSE06 | .892 | .022 | 40.55 | < .001 | .849 | .935 | .795 |
| VSE07 | .884 | .024 | 36.64 | < .001 | .837 | .931 | .781 |
| VSE08 | .859 | .026 | 32.94 | < .001 | .808 | .910 | .737 |
| VSE09 | .854 | .028 | 30.26 | < .001 | .799 | .909 | .729 |
| VSE10 | .874 | .025 | 35.01 | < .001 | .826 | .923 | .765 |
| VSE11 | .694 | .049 | 14.24 | < .001 | .598 | .789 | .481 |
| VSE12 | .696 | .051 | 13.64 | < .001 | .596 | .796 | .484 |
| VSE13 | .855 | .028 | 30.79 | < .001 | .801 | .910 | .732 |
| VSE14 | .897 | .024 | 37.23 | < .001 | .850 | .945 | .805 |
| VSE15 | .853 | .029 | 29.55 | < .001 | .796 | .909 | .727 |
| VSE16 | .822 | .033 | 24.76 | < .001 | .757 | .887 | .675 |
| VSE17 | .894 | .026 | 34.80 | < .001 | .843 | .944 | .799 |

Note. Std. estimate = standardised factor loading; Std. error = robust standard error; CI = confidence interval; R² = proportion of indicator variance explained by the latent factor. The prespecified, unmodified 17-item one-factor FSE and VSE models were estimated separately using the weighted least squares mean- and variance-adjusted (WLSMV) estimator for ordered-categorical indicators (N = 128). No correlated residuals or post hoc model modifications were included. All factor loadings were statistically significant at p < .001. Nevertheless, the item-level loading estimates should be interpreted together with the global model-fit indices and do not independently establish adequate structural validity, particularly for the VSE-17 model.
