# Supplementary material for: Cross-cultural adaptation and initial psychometric evaluation of the fruit and vegetable self-efficacy scales among secondary school adolescents in Selangor, Malaysia
Source: Front Public Health. 2026 Sep 29;14:1920784. doi: 10.3389/fpubh.2026.1920784 (PMC13658580; doi:10.3389/fpubh.2026.1920784)
Supplement: Supplementary file 9 [file Table_9.docx]

**Supplementary 9**

Table S9a. Exploratory item-set sensitivity analyses comparing the original 17-item and expanded 18-item FSE and VSE models.

| **Parameter** | **Fruit Self-Efficacy (FSE)** | | **Vegetable Self-Efficacy (VSE)** | |
| --- | --- | --- | --- | --- |
|  | **Original 17-item model** | **Expanded 18-item modelᵃ** | **Original 17-item model** | **Expanded 18-item modelᵇ** |
| Number of items | 17 | 18 | 17 | 18 |
| Model specification | Original source-item model | Original model + one exploratory context-specific item | Original source-item model | Original model + one exploratory context-specific item |
| Scaled χ² (df) | 190.973 (119) | 254.386 (135) | 396.605 (119) | 433.311 (135) |
| χ²/df | 1.60 | 1.88 | 3.33 | 3.21 |
| CFI | 0.974 | 0.960 | 0.952 | 0.953 |
| TLI | 0.970 | 0.955 | 0.945 | 0.947 |
| RMSEA (90% CI) | 0.069 (0.050–0.087) | 0.083 (0.068–0.099) | 0.136 (0.121–0.150) | 0.132 (0.118–0.146) |
| SRMR | 0.061 | 0.069 | 0.099 | 0.096 |
| Standardized factor-loading range | 0.647–0.862 | 0.715–0.856 | 0.694–0.897 | 0.684–0.912 |
| AVE | 0.622 | 0.626 | 0.708 | 0.712 |
| CR | 0.965 | 0.968 | 0.976 | 0.978 |

Note. CFA = confirmatory factor analysis; CFI = comparative fit index; TLI = Tucker–Lewis index; RMSEA = root mean square error of approximation; CI = confidence interval; SRMR = standardized root mean square residual; AVE = average variance extracted; CR = composite reliability; WLSMV = weighted least squares mean- and variance-adjusted. This table presents the exploratory item-set sensitivity analyses comparing the original 17-item and expanded 18-item FSE and VSE versions (N = 128). To ensure parallel comparisons, all four models were estimated using WLSMV as unmodified one-factor models without correlated residuals. The original 17-item VSE model presented here therefore differs from the refined primary VSE model, which included correlated residuals. AVE and CR were calculated from the standardized factor loadings and corresponding residual variances. All standardized factor loadings were statistically significant (p < .001). Because the models contained different sets of observed indicators and were not strictly nested, differences in fit indices were interpreted descriptively, and no formal χ²-difference testing was performed.

ᵃ The expanded FSE model included the exploratory context-specific item FSE-Add: “...buy fruit while undertaking leisure activities.”

ᵇ The expanded VSE model included the exploratory context-specific item VSE-Add: “...eat three or more servings of vegetables per day.” The labels FSE-Add and VSE-Add distinguish these exploratory items from the numbering of the final 17-item scales.

Table S9b. Leave-one-item-out sensitivity analysis of the original 17-item Vegetable Self-Efficacy Scale model (N = 128)

| **Omitted item** | **CFI** | **TLI** | **RMSEA** | **SRMR** | **Interpretation** |
| --- | --- | --- | --- | --- | --- |
| None: original 17-item model | 0.9517 | 0.9448 | 0.1355 | 0.0991 | Reference model |
| VSE01 | 0.9499 | 0.9422 | 0.1407 | 0.1009 | Worse fit |
| VSE02 | 0.9505 | 0.9428 | 0.1423 | 0.1041 | Worse fit |
| VSE03 | 0.9515 | 0.9441 | 0.1392 | 0.098 | No consistent improvement |
| VSE04 | 0.9528 | 0.9455 | 0.1403 | 0.1014 | No consistent improvement |
| VSE05 | 0.9516 | 0.9442 | 0.1408 | 0.1037 | Worse fit |
| VSE06 | 0.9479 | 0.9399 | 0.1424 | 0.1037 | Worse fit |
| VSE07 | 0.9534 | 0.9462 | 0.1354 | 0.0937 | Minor improvement |
| VSE08 | 0.9496 | 0.9418 | 0.1427 | 0.1044 | Worse fit |
| VSE09 | 0.9572 | 0.9506 | 0.1320 | 0.0891 | Partial improvement |
| VSE10 | 0.9594 | 0.9531 | 0.1270 | 0.0824 | Lowest SRMR; overall fit remained inadequate |
| VSE11 | 0.953 | 0.9458 | 0.1441 | 0.1035 | Worse fit |
| VSE12 | 0.9534 | 0.9462 | 0.1420 | 0.0998 | No consistent improvement |
| VSE13 | 0.9599 | 0.9537 | 0.1278 | 0.0948 | Partial improvement |
| VSE14 | 0.951 | 0.9435 | 0.1380 | 0.0978 | No consistent improvement |
| VSE15 | 0.9491 | 0.9413 | 0.1431 | 0.1043 | Worse fit |
| VSE16ᵃ | 0.9622 | 0.9564 | 0.125 | 0.0919 | Largest relative improvement; overall fit remained inadequate |
| VSE17ᵇ | 0.9524 | 0.9451 | 0.1362 | 0.0909 | Improvement in SRMR only |

**Note.** CFI, comparative fit index; TLI, Tucker–Lewis index; RMSEA, root mean square error of approximation; SRMR, standardized root mean square residual. Each source item was omitted separately, and the resulting 16-item unmodified one-factor model was re-estimated using weighted least squares mean and variance adjusted estimation (WLSMV), with residual covariances constrained to zero. The original unmodified 17-item model served as the reference model. Although omitting VSE16 produced the largest relative improvements in CFI, TLI, and RMSEA, and omitting VSE10 produced the lowest SRMR, no single-item deletion resulted in satisfactory overall model fit.

Table 9c. Sequential post hoc residual-covariance specifications for the VSE-17 model

| Model | Model 0 | Model 1 | Model 2 | Model 3 | Model 4 | Model 5 | Model 6 |
| --- | --- | --- | --- | --- | --- | --- | --- |
| Model specification | Original one-factor model without correlated residuals | Model 0 + VSE13 ↔ VSE16 | Model 1 + VSE09 ↔ VSE10 | Model 2 + VSE07 ↔ VSE10 | Model 3 + VSE07 ↔ VSE09 | Model 4 + VSE12 ↔ VSE16 | Model 5 + VSE14 ↔ VSE17 |
| χ² (df) | 396.605 (119) | 340.527 (118) | 311.050 (117) | 279.317 (116) | 260.413 (115) | 247.665 (114) | 238.507 (113) |
| χ²/df | 3.33 | 2.89 | 2.66 | 2.41 | 2.26 | 2.17 | 2.11 |
| CFI | 0.952 | 0.961 | 0.966 | 0.972 | 0.975 | 0.977 | 0.978 |
| TLI | 0.945 | 0.955 | 0.961 | 0.967 | 0.97 | 0.972 | 0.974 |
| RMSEA (90% CI) | 0.136 (0.121–0.150) | 0.122 (0.107–0.137) | 0.114 (0.099–0.130) | 0.105 (0.090–0.121) | 0.100 (0.084–0.116) | 0.096 (0.080–0.112) | 0.094 (0.077–0.110) |
| SRMR | 0.099 | 0.092 | 0.084 | 0.077 | 0.073 | 0.07 | 0.067 |

Note. CFA = confirmatory factor analysis; CFI = comparative fit index; CI = confidence interval; RMSEA = root mean square error of approximation; SRMR = standardized root mean square residual; TLI = Tucker–Lewis index; VSE = Vegetable Self-Efficacy; WLSMV = weighted least squares mean- and variance-adjusted estimator. Model 0 represents the prespecified one-factor VSE-17 model without correlated residuals. Models 1–6 represent sequential post hoc diagnostic specifications in which residual covariances were added individually based on modification indices, residual patterns, substantive similarities in item content, and theoretical plausibility. All specifications retained the original 17 items and were estimated using WLSMV for ordered-categorical indicators (N = 128). The residual covariances were identified and evaluated using the same sample. Although several fit indices changed across the sequence, the RMSEA remained above the prespecified criterion of 0.080 in Model 6, and acceptable global fit was not achieved. The statistical significance of the residual covariances was not interpreted as evidence of structural validity. These specifications are reported solely to document possible sources of local dependence and to generate hypotheses requiring evaluation in an independent sample; none should be regarded as a refined or validated measurement model.
